# Shared Numerosity Representations Across Formats and Tasks Revealed with 7 Tesla fMRI: Decoding, Generalization, and Individual Differences in Behavior

**DOI:** 10.1093/texcom/tgaa038

**Published:** 2020-07-30

**Authors:** Eric D Wilkey, Benjamin N Conrad, Darren J Yeo, Gavin R Price

**Affiliations:** Brain and Mind Institute, Western University, London, Ontario N6A5B7, Canada; Department of Psychology and Human Development, Peabody College, Vanderbilt University, Nashville, TN 37203, USA; Department of Psychology and Human Development, Peabody College, Vanderbilt University, Nashville, TN 37203, USA; Division of Psychology, School of Social Sciences, Nanyang Technological University, 639818, Singapore; Department of Psychology and Human Development, Peabody College, Vanderbilt University, Nashville, TN 37203, USA

**Keywords:** math achievement, multivoxel pattern analysis, number representation, numerical cognition, ultra-high field fMRI

## Abstract

Debate continues on whether encoding of symbolic number is grounded in nonsymbolic numerical magnitudes. Nevertheless, fluency of perceiving both number formats, and translating between them, predicts math skills across the life span. Therefore, this study asked if numbers share cortical activation patterns across formats and tasks, and whether neural response to number predicts math-related behaviors. We analyzed patterns of neural activation using 7 Tesla functional magnetic resonance imaging in a sample of 39 healthy adults. Discrimination was successful between numerosities 2, 4, 6, and 8 dots and generalized to activation patterns of the same numerosities represented as Arabic digits in the bilateral parietal lobes and left inferior frontal gyrus (IFG) (and vice versa). This indicates that numerosity-specific neural resources are shared between formats. Generalization was also successful across tasks where participants either identified or compared numerosities in bilateral parietal lobes and IFG. Individual differences in decoding did not relate to performance on a number comparison task completed outside of the scanner, but generalization between formats and across tasks negatively related to math achievement in the parietal lobes. Together, these findings suggest that individual differences in representational specificity within format and task contexts relate to mathematical expertise.

## Introduction

Working with numbers in a variety of representational formats is an important skill that children typically master early in development, and one that serves as a precursor to mathematical thinking (Dehaene 2011). Some representations of number are nonsymbolic, such as items in a set or beeps in a sequence, and are evident early in infancy (Izard et al. 2009). The perception of nonsymbolic number is likely rooted in an innate, evolutionarily ancient neural system that abstracts the property of numerical magnitude (i.e., the number of items) from continuous perceptual properties (i.e., object contours, overall surface area, density, etc.) (Feigenson et al. 2004; Dehaene 2011), though details of this system remain controversial (Leibovich et al. 2016; Knops 2017; Núñez 2017). Other representations of number are symbolic in nature, such as Arabic numerals or spoken words, and develop alongside language skills (Wiese 2003; Ansari 2008). There is currently no consensus view on whether the encoding of symbolic number is grounded in the nonsymbolic neural system (Piazza 2010), is developmentally independent, but may eventually be integrated (Carey et al. 2017; Carey and Barner 2019), or is perhaps linked at one point but then decoupled over developmental time (Lyons et al. 2012). Further, it is well-documented that individual differences in the fluency of perceiving both formats, and translating between them, is associated with superior mathematical skills across the life span (Iuculano et al. 2008; Mazzocco et al. 2011; Fazio et al. 2014; Price et al. 2017; Price and Wilkey 2017; Schneider et al. 2017; Wilkey et al. 2018), but what drives this relation is not well understood (for a critical review, see Wilkey and Ansari 2019).

Accordingly, several gaps in our current understanding of what drives individual differences in numerical processing remain. First, do symbolic and nonsymbolic representations of number share cortical patterns of activation? Second, are representations of number task-dependent? Third, do patterns of neural response to number relate to numerical ability?

### Shared Versus Independent Representation of Number Across Formats

A recent meta-analysis indicates that processing of nonsymbolic and symbolic number both activate bilateral regions of the parietal lobe and right frontal lobe (Sokolowski et al. 2017). Given the wide range of tasks, however, it is difficult to determine if this shared activity is attributable specifically to the processing of numerical magnitude, or other domain-general task-related features such as attention or response selection.

To address this problem, some studies have used adaptation paradigms that measure brain response during passive viewing rather than active, response-based tasks. This research has led to mixed evidence for either shared or distinct neural representation between number formats (Shuman and Kanwisher 2004; Piazza et al. 2007; Roggeman et al. 2007; Kadosh et al. 2011; Sokolowski et al. 2019). These mixed findings may be due to a reliance in this field on traditional univariate functional magnetic resonance imaging (fMRI) analytic approaches that require overlap of functional regions across participants in normalized space to reveal shared neural mechanisms across a sample. While this approach has proved informative, and univariate analyses conducted in subject-specific regions of interest (ROIs) or native space are becoming more common, it may be that the issue of shared versus distinct representations requires a more fine-grained approach that takes into account individual variability in cortical organization.

One alternative to the univariate group-averaging approach is to analyze patterns of activity across multiple voxels within an individual using multivariate pattern analysis (MVPA, Norman et al. 2006). Studies that have employed various types of MVPA analyses have provided conflicting evidence, with some showing evidence of cross-format classification (Eger et al. 2009; Teichmann et al. 2018; Bankson et al. 2019), particularly with smaller numbers (Damarla and Just 2013), while others suggest format-dependent patterns of neural activity (Bulthé et al. 2014, 2015; Lyons et al. 2015; Lyons and Beilock 2018; Sokolowski et al. 2019).

The reasons for the contradictory findings and the consequent lack of consensus, however, remain unclear. It is possible that relatively low sample sizes have increased variability in findings, or that the signal-to-noise ratio afforded by 3 Tesla fMRI is nonoptimal for detecting subtle spatial activation patterns. The current study addresses these 2 issues by collecting fMRI data at 7 Tesla (which increases the signal-to-noise ratio of the BOLD response, Yacoub et al. 2001; van der Zwaag et al. 2009; De Martino et al. 2011, 2008) with a larger sample size (*n* = 39).

### Shared Versus Independent Representation of Number Across Tasks

Beyond the issue of shared representation across formats, another outstanding and previously unexplored question is—are neural representations of number task-dependent? Depending on the scenario, numerical information may be acted upon in very different ways (e.g., using nominal, ordinal, or cardinal properties of number), and it remains unclear whether the same neural representations of number are engaged across differing task contexts. Some behavioral research suggests that representations of number are task-dependent. For example, the numerical distance effect (Moyer and Landauer 1967), whereby numbers further apart in magnitude are more easily compared than numbers that are closer together, is a common property of comparing numbers. In one study comparing task-dependent numerical properties, the distance effect was evident in symbolic number comparison tasks, but not in a visual numeral matching task (Goldfarb et al. 2011). Similarly, the spatial-numerical associations of response codes (SNARC) effect are task-dependent. In a study that manipulated verbal or spatial working memory load during a parity judgment and magnitude comparison task, the SNARC effect disappeared in the parity judgment task under verbal load and disappeared in the comparison task under spatial load (van Dijck et al. 2009). Together, these results suggest that task context affects the way in which we process numbers.

There is also a background of neurological case studies that support task-dependent aspects of number processing. Studying 2 individuals with pure alexia, Cohen and Dehaene (1995) reported that number identification performance differed considerably depending on task demands. Both patients could name digits in the context of a simple naming task or when comparing numbers but frequently misidentified the same digits as operands of addition problems. However, it is still unknown how shared or distinct neural mechanisms that encode numerical information relate to different task behaviors and to what extent they are independent. To address this question, the current study employs 2 tasks, a number identification task and a number comparison task to investigate whether number-specific patterns of neural activation are generalizable across task contexts.

### Representation of Number and Numerical Ability

A dominant theory in the field suggests that precision of numerical magnitude representations is directly related to the development of math skills (Butterworth 2005; Halberda et al. 2008a; Dehaene 2011; Wilkey et al. 2017; Wilkey and Price 2018). While a large body of behavioral research has investigated this link between performance on basic number processing tasks, such as the number comparison task, and individual differences in math abilities, there is a high degree of inconsistency in results across studies (for meta-analyses, see Chen and Li 2014; Fazio et al. 2014; Schneider et al. 2017, 2018). This inconsistency may, in part, be driven by variations in the myriad factors that influence performance on any given cognitive task. Neuroimaging, and in particular MVPA, offers the potential to investigate number-specific representational precision more directly.

To date, 2 fMRI studies have demonstrated a relation between neural responses to numerical magnitudes and behavioral measures of nonsymbolic numerical processing acuity. In a sample of 3–4-year-old children, Kersey and Cantlon (2017) found that neural tuning curves in the right intraparietal sulcus (IPS) predicted discrimination sensitivity in a nonsymbolic number comparison task. In adults, Lasne et al. (2018) showed that MVPA decoding performance classifying nonsymbolic numerosities correlated with individual differences in behavioral measures of nonsymbolic numerical acuity. The extent to which these results hold true for symbolic numbers, or to which behavioral performance is related to cross-format generalization, is unknown. To address this, the current study conducts a similar analysis as Lasne et al. (*n* = 12) with a larger sample (*n* = 39) assessing the relation between neural representations of nonsymbolic and symbolic formats and behavioral number comparison performance. We further explore whether decoding accuracy within each format relates to math achievement. If representational acuity of number does underlie math skill development, we should expect higher classification accuracy rates to correlate with higher math ability.

In regard to format generalization and math ability, Bulthé et al. (2018) reported that the degree of representational overlap, as indexed by MVPA generalization, between symbolic and nonsymbolic number in the parietal lobe negatively correlated with arithmetic ability. Such findings support the idea that with increasing expertise in symbolic numerical abilities, such as arithmetic, the neural systems used to represent symbolic number decouples from, or becomes “estranged” from, nonsymbolic representation (Lyons et al. 2012). However, Bulthé et al. limited their analysis to a combined left and right parietal ROI. Questions remain, therefore, about whether this pattern holds true for left and right parietal regions independently, and whether it can also be observed in frontal and temporal regions associated with the representation and processing of numerical information.

### The Current Study

In summary, to address the 3 questions outlined above, we use 7 Tesla fMRI to assess (1) whether patterns of neural response to specific numerical magnitudes in one format can generalize to the other, (2) whether patterns of neural response can generalize across tasks (i.e., number identification to number comparison), and (3) whether precision of neural representation is related to behavioral outcomes in basic number processing and math performance.

## Materials and Methods

### Participants

Forty neurologically healthy, right-handed individuals (screened via self-report) participated in the study for undergraduate course credit. Of those recruited, one participant was excluded from analyses due to poor data quality (see Data Quality Assessment), resulting in final sample of 39 participants (Mean age = 19.8 years, Range = 18.4–22.3, 20 females). All participants had normal or corrected-to-normal vision. Informed consent was obtained from each participant in accordance with the Institutional Review Board policy. A portion of the neuroimaging data (i.e., the *Compare* task) has been reported on previously with a different analytic method and study goal (Conrad et al. 2020).

### Procedure

The study consisted of 2 testing sessions, a behavioral session conducted in a quiet room and an MRI session conducted at the university’s imaging center. In the first session, participants completed a battery of academic, intelligence, and cognitive measures including a single-digit and double-digit symbolic number comparison task (only the single-digit task was analyzed since it was most comparable with the fMRI task), a nonsymbolic number comparison task, 2 math subtests of the Woodcock Johnson-III, a forward and backwards versions of the Corsi digit-span, and the Kaufman Brief Intelligence Test (second Edition). fMRI was acquired on the participants’ second session as soon as possible thereafter (Mean time between sessions = 7.9 days, Range = 1–28). All computer-based tasks were presented using *E*-Prime 2.0 (Psychology Software Tools). Preregistration of our analytic approach is archived here: https://osf.io/9uz72.

### Behavioral Assessment

#### Nonsymbolic Number Comparison

Participants were presented with 2 sets of dots simultaneously and asked to indicate via button press which set was more numerous (i.e., which set contained more dots). The set on the left side of the screen contained yellow dots and the set on the right side contained blue dots, which corresponded to color-coded left and right buttons, on a gray background. Response side was fully counterbalanced. Trials consisted of 1000 ms stimulus presentation followed by 2000 ms of a fixation cross. Seven ratios were presented, ranging from 0.33 (5 vs. 15) to 0.9 (9 vs. 10), for further details, see Supplementary Table S1. The number of dots in each stimulus ranged from 5 to 15. Each ratio was presented 10 times for a total of 70 trials. Ratios, stimulus presentation times, and order of presentation were modeled after Odic et al. (2014). To control for the possibility that participants might choose a strategy based on visual cues rather than number of dots, the following visual properties of dot sets were varied using a modified version of the MATLAB code recommended by Gebuis and Reynvoet (2011) to generate stimuli: convex hull (area extended by a stimulus), total surface area (aggregate value of dot surfaces), average dot diameter, and density (convex hull divided by total surface area). In approximately, one quarter of the trials all 3 visual properties were congruent with greater numerosity (i.e., the greater number of dots had a greater convex hull, surface area, etc.). In another approximate quarter of the trials, all 4 visual properties were incongruent with greater numerosity. In the remaining trials, visual properties were mixed congruent and incongruent. All stimuli were presented on a 21.5″ monitor driven at a refresh rate of 60 Hz and resolution of 1920 × 1080 pixels also using *E*-Prime 2.0. The 47.7 × 26.8 cm screen subtended 44.7° × 26.0° at the viewing distance of about 58 cm. The arrays of dots centered at 12.6° left and right of the center fixation point. Dot arrays were presented within square 506 × 506 pixel images (8.35° × 8.35°). The average dot diameter was 36.3 pixels (0.62°), the minimum dot diameter was 22.5 pixels (0.39°), and the maximum dot diameter was 56.8 pixels (0.97°). Further details of the visual parameters of the dot set (i.e., area subtended, surface area, diameter, and circumference of each dot array) can be found on the project page on the Open Science Framework: https://bit.ly/30A8Nj3.

To capture participants’ performance on the symbolic and nonsymbolic number comparison tasks, we adopted Lyons et al.’s (2014) performance metric *P* = RT(1 + 2ER), where RT is response time in milliseconds and ER is error rate. This metric expresses response time adjusted for error rate, such that response time is unchanged for students without errors, and response time is doubled for students who perform at chance (i.e., 50% error rate). Accordingly, a greater *P* score represents worse performance. The performance metric affords one outcome that combines response time and accuracy, and it adjusts for speed-accuracy trade-offs. We calculated error rate using all trials; to calculate mean response time we used correct trials, excluding outlier trials that were ±3 standard deviations from each student’s average response time. ﻿We also computed a second metric to index performance that is more closely related to previous analyses of the nonsymbolic number comparison task, ‘weber fraction’(*w*). *w* is derived from the Weber–Fechner law and is a metric of the noise in an individual’s representation of numerical magnitude. To compute our *w* scores, we used the method and formula employed by Halberda et al. (2008a). The percentage correct on the ANS task was modeled for each individual subject as 1—error rate, where error rate is defined as: $\frac{1}{2}\mathit{\operatorname{erfc}}\Big(\frac{n_1-{n}_2}{\sqrt{2w}\sqrt{n_1^2+{n}_1^2}}\Big)$, where erfc(*x*) is the complementary error function related to the integration of the normalized Gaussian distribution. The model fits percentage correct as a function of the Gaussian approximate number representations for the 2 sets displayed on a trial (*n*1 and *n*2) with a single free parameter for *w*.

#### Symbolic Number Comparison

Participants were simultaneously presented with single-digit Arabic numerals and asked to indicate via button press which of the 2 was numerically larger (e.g., 7 is larger than 6). The ratios presented, order of ratios, and stimuli durations were identical to those in the nonsymbolic number comparison task. Numerals ranged from 2 to 9. For further details, see Supplementary Table S1. Arabic digits were presented in Courier New font in light gray (i.e., “silver” in E-Prime) on a black background. Like the nonsymbolic stimuli, digits were presented at 12.6° left and right of center fixation, but were 72 × 132 pixels (1.23° × 2.25°) in size, on average.

#### Mathematics Achievement

Mathematical competence was assessed using the math fluency and calculation subtests of the Woodcock-Johnson III Tests of Achievement (WCJ-III) (Woodcock et al. 2001). The Math Fluency subtest requires participants to answer as many simple addition, subtraction, and multiplication problems as possible within a 3-min period. The calculation subtest, on the other hand, is untimed, and requires participants to complete as many calculation items as possible that increase in difficulty, ranging from simple arithmetic to calculus. A weighted, composite calculation skills cluster score comprising both subtests was computed for each participant using the WCJ scoring software. Grade-normed standard scores were used for all analyses. A Shapiro–Wilk test of normality demonstrated that the math measure was not normally distributed (*P* = 0.016), with a negative skew of −0.855 (se = 0.378). Therefore, in order to conduct correlational analyses that assume a normal distribution of measures, we squared the standard scores which resulted in a normally distributed sample of abilities (Shapiro–Wilk *P* = 0.159).

### MRI Session

#### MRI Acquisition Parameters

Imaging was performed using a 7 Tesla (7 T) Philips Achieva scanner with a 32-channel head coil. An MP2RAGE (Marques et al. 2010) image was acquired for anatomical reference, aligned to the anterior/posterior commissures, with the following parameters: TR = 4.315 ms, TE = 1.92 ms, flip angle = 7, 240 coronal slices, voxel size = 1 mm^3^, imaging matrix = 240 x 240 x 192, acquisition time = 1010 s. These images were corrected for B1-field inhomogeneities, as well as proton density and T2* effects according to the procedure described by Marques et al. (2010). For the event-related experiment, functional T2*-weighted images were acquired over 2 runs of 243 volumes each, with the following parameters: TR = 2000 ms, TE = 25 ms, flip angle = 63, 46 axial slices (with no interslice gap), voxel size = 2.5 mm^3^, imaging matrix = 96 x 96 x 46, acquisition time = 500 s per run (33:20 m of functional data total).

#### fMRI Tasks

Participants completed in order: a scout scan, 2 runs of an event-related number identification paradigm (*Identify*), an anatomical scan, and then 2 consecutive runs of an event-related number comparison paradigm (*Compare*). Tasks were not counter-balanced because we anticipated that completing the comparison task first may induce a lasting cognitive effect to automatically assess the quantity and compare it with the standard. Accordingly, participants always completed the *Identify* task first. Further, as our analysis plan involved individual differences, varying the task order across participants may introduce irrelevant variance in our measures of interest.

**Figure 1:**
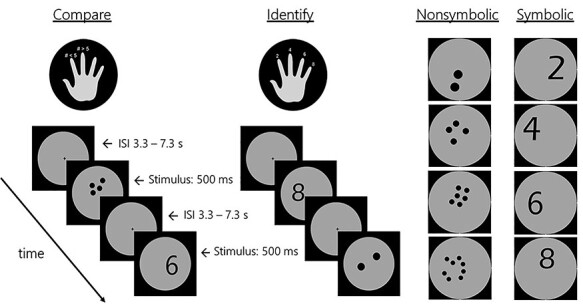
FMRI task paradigms and stimulus examples for all 4 numerical magnitudes in both formats.

***Identify.*** For each trial, participants judged whether an Arabic digit (“symbolic”) or dot array (“nonsymbolic”) could be identified as 2, 4, 6, or 8 by pressing one of 4 buttons on their right hand as quickly and accurately as possible, Fig. 1. A total of 160 trials were presented, composed of 80 symbolic trials and 80 nonsymbolic trials (20 per number, per format) which were intermixed and pseudorandomly ordered (i.e., no more than 3 consecutive trials were of the same number and same format). Nonsymbolic stimuli were created using the MATLAB package first described by Piazza et al. (2004). Nonsymbolic stimuli were controlled for total surface area across numerosities by reducing dot size with increasing numerosity. Additionally, all stimuli were controlled for total occupied area and luminance across formats (i.e., on average, dots sets contained the same number of pixels as Arabic digits) in an effort to control for non-numerical visual parameters across trials. Dot sets and digits were presented in black [RGB: 0, 0, 0] on a gray background [RGB: 180, 180, 180] encircled by a black border. Location within the gray circle varied across trials but was balanced for quadrant between all conditions. Stimulus duration was 500 ms and interstimulus intervals (ISI) ranged from 3300 to 7300 ms, in 1000 ms increments, with an average of 5300 ms. ISI was counterbalanced across numerosities and conditions.

***Compare.*** The same stimuli were used for the “compare” condition, except in this task, participants were instructed to indicate whether the number they saw was less than or more than 5 by pressing a button with either their right index or right middle finger, respectively.

### MRI Data Processing

#### fMRI Preprocessing

FMRI data were preprocessed in AFNI using the afni_proc.py program, including despiking, slice-time and motion correction, coregistration, normalization to a MNI152 template, and scaling (Cox 1996). No spatial smoothing was applied. Participant-level activation analyses to estimate the effect of all trials versus baseline were carried out using 3dREMLfit, which accounts for time series autocorrelation. Baseline regressors included 6 motion parameters and their derivatives, and zeroth- to fourth-order Legendre polynomials to model low-frequency drifts (per run).

#### Machine Learning Methods

MVPA decoding and generalization pattern classification were implemented in MATLAB (Mathworks, Natick, MA) using the linear discriminant analysis (LDA) classifier in the CoSMo MVPA toolbox (cosmomvpa.org, Oosterhof et al. 2016). Statistics were conducted in R (R Core Team 2018) using the ggplot2 (Hadly Wickham 2016), vioplot (Adler and Kelly 2018), and tidyverse packages (Wickham 2017) for graphical display and data handling as well as jamovi (2019, https://www.jamovi.org, version 0.9).

#### Preprocessing Betas for Classification

Per-trial beta maps (i.e., activation maps) were created using a second, participant-level GLM and estimated with AFNI’s 3dDeconvolve function. Separate regressors were included for each of the 320 trials, modeling trial-wise BOLD responses, as well as all nuisance regressors described above (Rissman et al. 2004). As a final step, to ensure that potential differences in activation magnitude between tasks (i.e., identify vs. compare) did not confound pattern classification across tasks (or within tasks, across runs), we implemented a spatial normalization procedure involving subtraction of the voxel-wise mean and division by the voxel-wise standard deviation, across voxels within each ROI (Misaki et al. 2010). In other words, we *z*-normalized each set of voxel-wise betas at the trial level. The resulting series of normalized beta vectors were sorted by condition and served as inputs for subsequent MVPA’s. Each per-trial beta map is considered a sample in the analysis.

#### Regions and Voxels of Interest

MVPA classification analysis was conducted in the 8 regions of interest, 4 regions of interest per hemisphere. Inferior frontal and parietal regions were chosen due to the convergence of evidence across seminal works and meta-analyses that they are involved in numerical magnitude representations (Arsalidou and Taylor 2011; Arsalidou et al. 2017; Sokolowski et al. 2017). Recent work, including meta-analyses has converged on the presence of a “number form area” (NFA) located in the posterior (Yeo et al. 2017), inferior temporal lobe that is integral for processing Arabic numerals and may relate to individual differences in math achievement (Pollack and Price 2019), so this region was also selected. Lastly, based on evidence from electrocorticography studies that show the coupling between parietal regions and inferior temporal regions during number-related tasks (Daitch et al. 2016; Baek et al. 2018), we hypothesized that the NFA and parietal mechanisms may reveal patterns together that provide more information than either region independently. To explore neural patterns of number representation as the 2 ROIs function together, we created an ROI that was the combination of our selected parietal region and the NFA region. If spatial patterns of activation span the 2 regions in a way that provides more number-specific decoding information, the “NFA + parietal” region should have significantly higher classification accuracy rates than either region independently.

**Figure 2:**
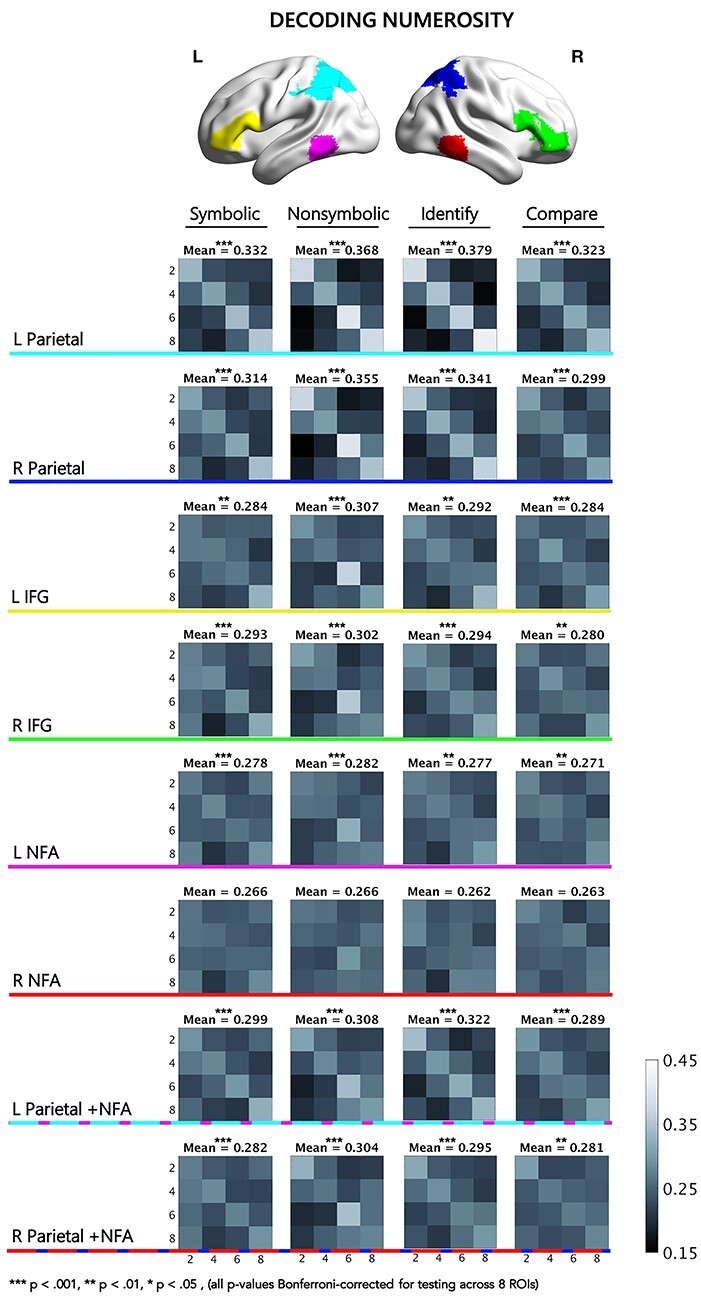
Numerosity decoding within formats and tasks. Confusion matrices for MVPA classification in 8 regions of interest used for MVPA classification averaged across participants. Mean = average classification across numerosities (diagonal squares); *x*-axis = predicted values; *y*-axis = target values; L = Left; R = Right; IFG = inferior frontal gyrus; NFA = number form area. Color bar represents classification rate as a percentage.

Regions were defined as follows: (1) the inferior frontal gyrus (IFG) (left and right), (2) the parietal lobe (left and right), (3) the NFA (left and right), and (4) the combination of the NFA and the parietal lobe ROIs (left and right) (Fig. 2). Anatomical masks for the IFG and the parietal region were derived from the WFU PickAtlas (Maldjian et al. 2003, 2004). Parietal ROIs were formed from combining the “superior parietal lobule” and “inferior parietal lobule”, and “inferior frontal gyrus” was selected for the IFG, split by hemisphere. The right NFA ROI was defined from the Yeo et al. meta-analysis (2017) by creating a spherical ROI with a 10 mm radius centered at the peak coordinate of convergence in the meta-analysis. The left NFA ROI was defined as the mirrored homologue of the right NFA ROI. To reduce features, a contrast of all stimuli versus implicit baseline was run and voxel-wise maps of *t*-statistics for each participant were computed. Within each ROI, we selected the 600 most significantly active voxels based on the highest *t*-statistics from the all versus baseline contrast as in Lasne et al. (2018). When the NFA and Parietal ROIs were combined, we selected 300 voxels with the highest *t*-statistics in this contrast from each region (600 total). We should note that because this contrast involves all conditions, no condition-specific selection bias is involved in the selection of these voxels. The feature-selection and classification analyses are sufficiently independent, a fact that was supported by the random permutation testing we conducted.

#### Data Quality Assessment

To validate that the current data were of sufficient quality and sensitivity to enable our MVPA classification analyses, we conducted an analysis of button presses in a spherical ROI of 1200 voxels (2.5 mm^3^) in the M1 motor strip on the precentral gyrus, corresponding to neurosynth.org’s peak *t*-statistic for the search term “finger movement” (MNI coordinates: −36, −28, 52). Training and testing conditions were separable button responses (separate fingers, all 4 fingers) with 20 trials per finger using a leave-one-out cross-validation technique with data on the identify condition. Twenty trials represent the minimum number of trials we expected to run our classifier on in the main analysis. If a participant did not have above-chance classification according to separate finger movements in a cortical location with a well-known spatial topography related to motor control, then data were not expected to be valid for classification of higher-level cognitive processes. According to this criterion, only one participant did not demonstrate above-chance classification in the motor regions. Upon inspection, this participant did have a considerable amount of movement during data collection. Therefore, the one participant for whom this was the case was excluded from further analyses. To make sure more fine-grained movements did not affect our analyses, we checked to see if overall movement correlated with classification accuracy rates by correlating movement with the classification accuracy rates in the M1 ROI. Movement was indexed by flagging volumes that demonstrated between-volume movement of >0.3 mm Euclidian norm distance or if >5% of voxels within a brain mask were determined to be outliers (signal > 5.5 median absolute deviations). Results indicated no significant correlation between number of flagged volumes and classification accuracy rate [*r* (37) = −0.089, *P* = 0.588].

### Analyses

#### Decoding

Before asking if patterns of neural activity generalized across formats or tasks, we needed to establish that the LDA classifier implemented in the current study could accurately decode the numerosity of a stimulus within the same format and within the same task. Therefore, the first step was to decode the 4 numerosities (2, 4, 6, and 8) within each condition (format x task) using trial-level beta maps (voxelwise maps per trial derived from event-related design). This resulted in four, 4 x 4 decoding/confusion matrices for each ROI. Higher decoding accuracies indicate more discriminable patterns of activation. ﻿Decoding accuracies were then averaged over numerosities to attain a single classification accuracy pertaining to conditions of interest (i.e., mean accuracy for symbolic, nonsymbolic, identify task, and compare task).

For all classification in the current study, we followed the same procedure. We followed a leave-three-out, cross-validation procedure where the classifier was trained on all but 3 sets of trial-level beta maps (set = one beta map per numerosity, or “chunk” in CoSMo’s terminology) in order to keep the number of training samples and test samples balanced. All possible combinations of training samples for left-out sets were used. For example, when decoding Symbolic number, where there were 40 trials per number, 3 trials of each number were left out for training, leaving 37 trials of each condition to train on, and 3 of each to test on (i.e., leave-three-out). Classification results were tested for significance (*P* < 0.05) across participants with a 2-tailed *t*-test, testing against the null of a chance-level classification (25%, given the 4 numerosities). All reported decoding *P-*values resulting from the *t*-tests against chance are Bonferroni-corrected by multiplying the uncorrected *P-*value by the number of ROIs for that test (*n* = 8). All classification results were examined for bias by random permutation tests (1000 permutations) for each analysis. In this process, the labels for training the LDA classifier are scrambled at each iteration, and, if the algorithm is unbiased, it should produce a normal distribution of classification accuracy centered around chance (25%). For all of the classifications in the current study, the mean of the deviation from 25% was negligible, indicating no bias in our algorithm. The permutation testing is reported with our data, but is not analyzed further.

In short, a result of numerosity decoding significantly above 25% averaged across numerosities and across individuals would indicate that, on average, neural activity in the ROI contains information related specifically to numerical magnitudes. Statistical tests are reported as one-sample, 2-tailed *t*-tests where the null being tested is a chance rate of decoding (25%).

#### Generalization

Our first 2 questions of interest, regarding shared neural representation for number between (1) numerical format and (2) task were addressed by testing whether classifiers can train on one format or task and generalize to the other. If the classifier can generalize number classification from one condition to the other, and there is no other alternative explanation for shared neural activity between numbers such as response selection or another confound, then the 2 formats (or tasks) can be assumed to share numerosity-specific patterns of neural activity. The same general procedures were used to test generalization as were used for decoding, except, rather than remove sample sets in an *n*-fold fashion, the classifier was trained on all samples of one condition and tested on all of the samples of the other. Therefore, rather than average over the thousands of *n*-fold test combinations, classifier performance within an individual is the mean number of correct predictions per condition. The same classifier, statistical tests, and random permutation testing were used for classification and generalization. All reported *P-*values for the *t*-test against chance classification are Bonferroni-corrected.

#### Classification–Behavior Correlation Analyses

Our third question of interest was whether patterns of neural response to number relate to (a) number comparison performance, and (b) math achievement.

To examine if individual differences in numerosity decoding predicted number comparison performance, a common measure of numerical acuity, we ran bivariate correlations between each participants’ mean within-format decoding classification accuracy rate (i.e., averaged across numerosities 2, 4, 6, and 8) and the behavioral performance metric for each participants’ performance in the number comparison task completed outside of the scanner. Correlations were run within formats. For example, decoding accuracy for nonsymbolic stimuli was correlated with performance on the nonsymbolic number comparison task.

Next, we investigated if decoding accuracy rates correlated with math achievement. Mean decoding classification accuracy rates for both nonsymbolic and symbolic stimuli were correlated with grade-normed standard scores of math achievement that had been squared to achieve a normal distribution.

Lastly, using bivariate correlations we tested whether participant’s mean format generalization values (from symbolic to nonsymbolic and vice-versa, averaged together) and mean task generalization values (from Identify to Compare and vice-versa, averaged together) correlated with math achievement, again squared.

In order to more directly compare with significance level of previous studies that ran similar correlations with various numbers of tests, none of the *P-*values for brain–behavior correlations are corrected for multiple comparisons.

## Results

### Decoding

#### Within-Format Numerosity Classification

Classification accuracy rates for nonsymbolic numerosities were above chance in 7 of the 8 ROIs (Bonferroni-adjusted *p*-value reported) (see Fig. 2 for means): L parietal [*t*(38) = 12.06, *P* < 0.001], R parietal [*t*(38) = 10.21, *P* < 0.001], L IFG [*t*(38) = 6.77, *P* < 0.001], R IFG [*t*(38) = 5.54, *P* < 0.001], L NFA [*t*(38) = 4.78, *P* < 0.001], R NFA [*t*(38) = 2.23, *P* = 0.056], L parietal and NFA [*t*(38) = 7.13, *P* < 0.001], R NFA and parietal [*t*(38) = 6.04, *P* < 0.001]. Only decoding in the right NFA failed to show above-chance classification accuracy. This indicates that in 7 of 8 ROIs there were distinguishable neural patterns for nonsymbolic stimuli of different magnitudes. These data are in overall agreement with the decoding accuracies obtained in previous research in the parietal lobe (Eger et al. 2009; Bulthé et al. 2015) and frontal regions (Bulthé et al. 2014). Comparing classification rates in parietal ROIs versus parietal + NFA ROIs indicated that including the NFA with parietal data had a significant negative impact on classification accuracy [left: *t*(38) = 5.50, *P* < 0.001, Cohen’s *d* = 0.88; right: *t*(38) = 5.50, *P* < 0.001, Cohen’s *d* = 0.88], indicating that parietal ROIs carried all of the important information about numerosity-specific processing in the combined ROI. Therefore, the combined parietal + NFA ROIs are not analyzed further in the classification–behavior correlations.

Decoding of symbolic numerosities followed the same pattern of results as nonsymbolic stimuli. Classification accuracy rates for symbolic numerosities were above chance in 7 of the 8 ROIs (Bonferroni-adjusted *p*-value reported) (see Fig. 2 for means): L parietal [*t*(38) = 11.00, *P* < 0.001], R parietal [t(38) = 7.64, *P* < 0.001], L IFG [*t*(38) = 4.03, *P* = 0.002], R IFG [*t*(38) =5.05, *P* < 0.001], L NFA [*t*(38) = 4.32, *P* < 0.001], R NFA [*t*(38) = 2.41, *P* = 0.167], L parietal and NFA [*t*(38) = 5.72, *P* < 0.001], R NFA and parietal [*t*(38) = 4.47, *P* < 0.001]. Only decoding in the right NFA failed to show above-chance classification accuracy. Again, this indicates that in 7 of 8 ROIs there were distinguishable neural patterns for symbolic stimuli of different numerosities. Comparing classification rates in parietal ROIs versus parietal + NFA ROIs indicated that including the NFA with parietal data had a significant negative impact on classification accuracy [left: *t*(38) = 4.39, *P* < 0.001, Cohen’s *d* = 0.70; right: *t*(38) = 3.46, *P* = 0.001, Cohen’s *d* = 0.55], indicating that parietal ROIs carried all of the important information about task generalization. Therefore, the combined parietal + NFA ROIs are not analyzed further in the classification–behavior correlations.

For detailed plots of means and ranges of decoding performance within conditions across numerosities, see Supplementary Figure S1.

#### Within-Task Numerosity Classification

Mean classification accuracy rates for numerosities in the identify task collapsed across formats were above chance in 7 of the 8 ROIs (Bonferroni-adjusted *p*-value reported) (see Fig. 2 for means): L parietal [t(38) = 13.03, *P* < 0.001], R parietal [*t*(38) = 9.06, *P* < 0.001], L IFG [*t*(38) = 6.37, *P* < *P* < 0.001], R IFG [*t*(38) = 5.33, *P* < 0.001], L NFA [t*(*38) = 3.95, *P* = 0.003], R NFA [*t*(38) = 1.70, *P* = 0.778], L parietal and NFA [*t*(38) = 7.47, *P* < 0.001], R NFA and parietal [*t*(38) = 5.40, *P* < 0.001]. As above, only decoding in the right NFA failed to show above-chance classification accuracy, indicating that in 7 of 8 ROIs, there were distinguishable neural patterns for stimuli of different numerosities within the identify task across numerical formats.

Decoding of numerosities in the Compare task followed the same pattern of results as in the Identify task, albeit with somewhat lower mean accuracy rates. Classification accuracy rates for numerosities in the Compare task were above chance in 7 of the 8 ROIs (Bonferroni-adjusted *p*-value reported) (see Fig. 2 for means): L parietal [*t*(38) = 9.04, *P* < 0.001], R parietal [*t*(38) = 6.18, *P* < 0.001], L IFG [*t*(38) = 5.03, *P* < 0.001], R IFG [*t*(38) = 3.93, *P* = 0.003], L NFA [*t*(38) = 3.84, *P* = 0.004], R NFA [*t*(38) = 1.71, *P* = 0.762], L parietal and NFA [*t*(38) = 4.78, *P* < 0.001], R NFA and parietal [*t*(38) = 4.08, *P* = 0.002]. Only decoding in the right NFA failed to show above-chance classification accuracy, indicating that in 7 of the 8 ROIs, there were distinguishable neural patterns for stimuli of different numerical magnitudes within the Compare task across numerical formats.

For detailed plots of means and ranges of decoding performance within conditions across numerosities, see Supplementary Figure S2. For within-task, within-format classification accuracy rates, see Supplementary Tables S2–S5.

### Generalization

#### Generalization Between Numerical Formats

**Figure 3:**
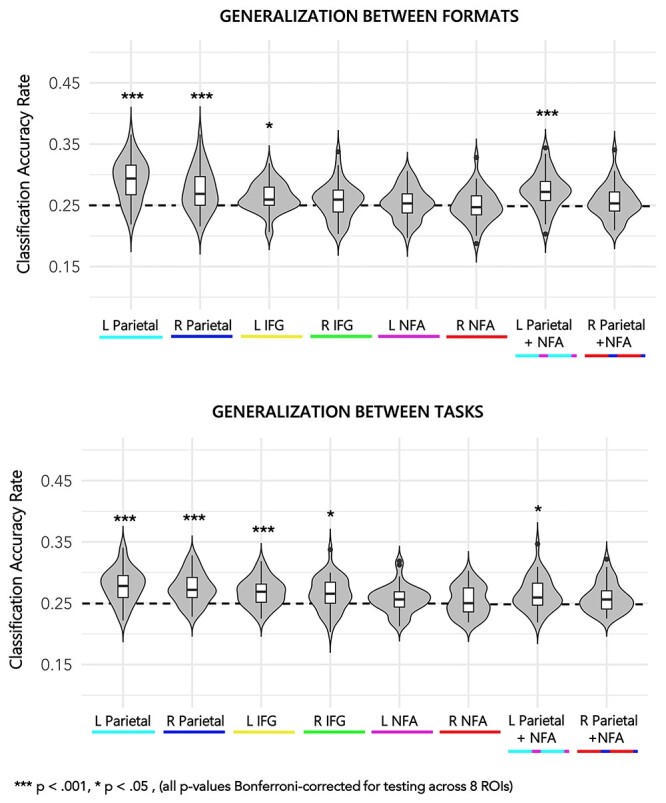
Generalization of activation patterns for numerosities between formats (top) and between tasks (bottom). Color of ROIs corresponds to brain map in Figure 2. Classification accuracy rate = average classification across numerosities within ROI; L = left; R = right; IFG = inferior frontal gyrus; NFA = number form area. Box plot hinges represent 25th and 75th percentile of distributions, whiskers extend from hinge to the largest value not beyond 1.5 times the interquartile range. All points plotted beyond whiskers. Dotted horizontal line = classification accuracy rate at chance (25%).

To test for shared patterns of activation during Symbolic and Nonsymbolic numerical stimuli, we tested if the classifier could train in one format and predict patterns of activation in the other. The following results collapse across tasks (i.e., assuming that there is some shared number-specific pattern because both tasks require common identification (visual and verbal encoding) processes) and take the average of training/testing in both the Nonsymbolic $\rightarrow$ Symbolic and Symbolic $\rightarrow$ Nonsymbolic directions. Mean classification accuracy rates were above chance in 4 of the 8 ROIs (Bonferroni-adjusted *p*-value reported) (see Fig. 3 for means and distributions): L parietal [*t*(38) = 7.47, *P* < 0.001], R parietal [*t*(38) = 4.46, *P* < 0.001], L IFG [*t*(38) = 3.34, *P* = 0.015], R IFG [*t*(38) = 1.42, *P* = 1.000], L NFA [*t*(38) = 0.77, *P* = 1.000], R NFA [*t*(38) = −0.18, *P* = 1.000], L parietal and NFA [*t*(38) = 4.65, *P* < 0.001], R NFA and parietal [*t*(38) = 1.20, *P* = 1.000]. Comparing classification rates in parietal ROIs versus parietal + NFA ROIs indicated that including the NFA with parietal data had a significant negative impact on classification accuracy [left: *t*(38) = 3.21, *P* = 0.003, Cohen’s *d* = 0.51; right: *t*(38) = 3.41, *P* = 0.002, Cohen’s *d* = 0.55], indicating that parietal ROIs carried all of the important information about task generalization. Therefore, the combined parietal + NFA ROIs are not analyzed further for format generalization in the classification–behavior correlations.

To ensure that above generalization results were not driven by generalization from one format to another unidirectionally, we also calculated generalization between numerical formats separated by direction (Nonsymbolic $\rightarrow$ Symbolic and Symbolic $\rightarrow$ Nonsymbolic). Results are reported in Supplementary Tables S6 and S7. The pattern of above-chance generalizations across ROIs is identical to the results averaged across directions with one exception, Nonsymbolic numerosities did not generalize to Symbolic numerosities in the left IFG (*P* = 0.146). Although it is valid to assume a shared number-specific pattern across tasks due to a common identification process, there could be a greater proportion of unshared than shared patterns as a function of the task, so we also investigated how the classifier performed generalizing between formats within each task to see if there were task-level differences, albeit with lower power (20 training trials per condition instead of 40). The primary difference of note in this analysis was that across-format generalization of numerosity-specific activation was limited to the L Parietal ROI in the Compare task (for both Nonsymbolic $\rightarrow$ Symbolic and Symbolic $\rightarrow$ Nonsymbolic), whereas generalization was above chance in both the L and R Parietal ROI in the Identify task (L parietal: Nonsymbolic $\rightarrow$ Symbolic and Symbolic $\rightarrow$ Nonsymbolic; R parietal: Symbolic $\rightarrow$ Nonsymbolic only). Taken together, task-related differences seem to be limited to the R Parietal ROI. Detailed results are reported in Supplementary Tables S8–S12. However, it should be mentioned that the primary analyses and supplementary analyses are not directly comparable due to (A) a considerable difference in power, and (B) the fact that training was collapsed across tasks in the primary analysis, which inherently means that the LDA classifier was trained on a broader set of cognitive factors.

#### Generalization Between Tasks

To test for shared patterns of activation for numerosities in the context of both a comparison task and an identification task, we tested if the classifier could train in one task and predict patterns of activation in the other. The following results collapse across numerical format (i.e., assuming that there is some shared number-specific pattern because both formats require common verbal-encoding processes) and take the average of both the Identify $\rightarrow$ Compare and Compare $\rightarrow$ Identify directions. Mean classification accuracy rates were above chance in 5 of the 8 ROIs (all Bonferroni-adjusted *P* < 0.05) (see Fig. 3 for means and distributions): L parietal [*t*(38) = 6.48, Bonferroni-adjusted *P* < 0.001], R parietal [*t*(38) = 5.87, *P* < 0.001], L IFG [*t*(38) = 4.87, *P* < 0.001], R IFG [*t*(38) = 3.37, *P* = 0.014], L NFA [*t*(38) = 1.74, *P* = 0.715], R NFA [*t*(38) = 1.06, *P* = 1.000], L parietal and NFA [*t*(38) = 3.46, *P* = 0.011], R NFA and parietal [*t*(38) = 2.42, *P* = 0.162]. As above, comparing classification rates in parietal ROIs versus Parietal + NFA ROIs indicated that including the NFA with parietal data had a significant negative impact on classification accuracy [left: *t*(38) = 3.36, *P* = 0.002, Cohen’s *d* = 0.54; right: (38) = 2.78, *P* = 0.008, Cohen’s *d* = 0.45], indicating that parietal ROIs carried all of the important information about task generalization.

As in the cross-format generalization analysis, to ensure that above generalization results were not driven by generalization from one task to another unidirectionally, we also calculated generalization between numerical formats separated by direction (Identify $\rightarrow$ Compare and Compare $\rightarrow$ Identify). Results are reported in Supplementary Tables S13 and S14. Results are similar, with no differences in parietal regions, but there were lateralization differences in the IFG. Whereas patterns of numerosity-related neural activity generalized from the Identify task to the Compare task in the L IFG (but not R IFG), the reverse was evident (Compare to Identify) in the R IFG (but not L IFG). Again, as with cross-format generalization, we also investigated how the classifier performed generalizing between tasks within each format to see if there were format-level differences. The primary difference of note in this analysis was that across-task generalization of numerosity-specific activation in the L and R IFG was limited to Nonsymbolic numerosities (L IFG: both Identify $\rightarrow$ Compare and Compare $\rightarrow$ Identify; R IFG: Compare $\rightarrow$ Identify only). Detailed results are reported in Supplementary Tables S15–S19. Again, it should be mentioned that the primary analyses and supplementary analyses are not directly comparable due to differences (A) power and (B) the fact that training was collapsed across formats in the primary analysis, which means that the classifier was trained on a broader set of cognitive factors that may be shared between number formats.

### Classification–Behavior Correlations

#### Decoding of Nonsymbolic Number and Nonsymbolic Number Comparison

Across the 6 ROIs investigated, no region showed a correlation between decoding accuracy of Nonsymbolic numerosities and performance (*P*) on the behavioral nonsymbolic number comparison task (Table 1). We had preregistered running the correlation with performance score in order to compare similar metrics across task formats and avoid poor-fitting Weber models in the symbolic task, since symbolic number comparison task accuracy rates typically suffer from ceiling effects. However, since previous studies have shown a significant correlation between decoding and nonsymbolic number comparison Weber fractions (Lasne et al. 2018), for the sake of comparison across studies, we replicated our analysis using Weber fractions and again found no significant correlations across any of the selected ROIs. To provide measurable evidence in support of both positive and null findings, we conducted complementary Bayesian correlations in jamovi using the jsq—Bayesian Methods package (version 0.9.2), and their default priors (stretched beta prior width = 1). We report the Bayes Factor (BF_01_), which indicates the likelihood that the evidence is in favor of the null hypothesis relative to the alternative hypothesis. For instance, a BF_01_ of 3 suggests that the data were 3 times more likely to occur under the null than the alternative hypothesis. BFs > 3, 10, 30, and 100 are considered “moderate,” “strong,” “very strong,” and “extreme” evidence in support of the null hypothesis (Wagenmakers et al. 2018). Bayes factors (Table 1) suggested mostly moderate support for the null hypothesis of no correlation between either of the nonsymbolic performance metrics and decoding accuracy of Nonsymbolic numerosities. The decision to include Bayes factors was made after finding mostly null results, which contrasted with previously published results using a smaller sample size (Lasne et al. 2018). In order to more directly compare to the significance level of previous studies that ran similar correlations with various numbers of tests, none of the *P-*values for brain-behavior correlations are corrected for multiple comparisons.

**Table 1:** Correlations between decoding accuracy and performance on independent, same-format number comparison task (e.g., mean symbolic decoding accuracy across numerosities ~ symbolic comparison *P*), *n* = 39

		Decoding accuracy rates					
Task performance		L Par	R Par	L IFG	R IFG	L NFA	R NFA
Nonsymbolic comparison *P*	Pearson *r*	0.073	0.093	−0.013	−0.103	−0.04	0.053
	*P-*value	0.657	0.575	0.936	0.533	0.809	0.750
	BF_01_	4.56	4.31	5.00	4.16	4.88	4.77
Nonsymbolic comparison *w*	Pearson *r*	0.066	−0.054	0.101	0.083	−0.014	−0.124
	*P-*value	0.688	0.745	0.541	0.617	0.93	0.453
	BF_01_	4.64	4.77	4.19	4.45	5.00	3.82
Symbolic comparison *P*	Pearson *r*	−0.185	−0.156	−0.156	−0.217	−0.018	0.018
	*P-*value	0.259	0.344	0.344	0.184	0.912	0.912
	BF_01_	2.71	3.26	2.08	2.14	4.99	4.99

Notes: BF_01_ = Bayes factor for Pearson’s *r* correlation indicating probability of support for the null hypothesis (less than 1 indicates support for alternative, greater than 1 support for the null).

#### Decoding of Symbolic Number and Symbolic Number Comparison

Using the same analytic approach described above, we tested for relations between neural decoding of Symbolic numbers and performance on the out-of-scanner symbolic comparison task. Similar to the Nonsymbolic analysis, none of the 6 ROIs showed a correlation between decoding accuracy and behavioral performance. Bayes factors suggested mostly moderate support for the null of no correlation between symbolic comparison performance and Symbolic numerosity decoding accuracy, although Bayes Factors < 3 in parietal and IFG ROIs should be interpreted as inconclusive evidence with the current sample size (Table 1).

#### Decoding of Number and Mathematics Achievement

We tested for a relation between neural representation of number and math achievement by correlating decoding accuracy rates for each ROI and number format with mathematics achievement scores. Across the 6 ROIs investigated, no region showed a correlation between decoding accuracy rates and math achievement scores for either format (Table 2). This was true when considering math achievement composite scores and when considering subtests individually (Supplementary Table S23). Again, due to a pattern of mostly null results, we explored the evidence in favor of the null by computing Bayes factors. Bayes factors suggested mostly moderate support for the null hypothesis of no correlation between math achievement and decoding performance.

**Table 2:** Correlations between decoding accuracy rates and measures of math achievement (e.g., mean symbolic decoding accuracy across numerosities ~ math achievement), *n* = 39

Nonsymbolic decoding accuracy		Math achievement	Symbolic decoding accuracy		Math achievement
L Parietal	Pearson *r*	−0.067	L Parietal	Pearson *r*	−0.036
	*P-*value	0.687		*P-*value	0.827
	BF_01_	4.64		BF_01_	4.90
R Parietal	Pearson *r*	0.183	R Parietal	Pearson *r*	−0.060
	*P-*value	0.266		*P-*value	0.717
	BF_01_	2.76		BF_01_	4.71
L IFG	Pearson *r*	−0.062	L IFG	Pearson *r*	0.173
	*P-*value	0.708		*P-*value	0.293
	BF_01_	4.69		BF_01_	2.94
R IFG	Pearson *r*	−0.086	R IFG	Pearson *r*	−0.067
	*P-*value	0.603		*P-*value	0.684
	BF_01_	4.40		BF_01_	4.63
L NFA	Pearson *r*	0.270	L NFA	Pearson *r*	0.179
	*P-*value	0.096		*P-*value	0.276
	BF_01_	1.32		BF_01_	2.83
R NFA	Pearson *r*	−0.102	R NFA	Pearson *r*	0.127
	*P-*value	0.535		*P-*value	0.440
	BF_01_	4.17		BF_01_	3.76

Notes: BF_01_ = Bayes factor for Pearson *r* correlation indicating probability of support for the null hypothesis (less than 1 indicates support for alternative, greater than 1 support for the null).

#### Cross-Format Generalization and Mathematics Achievement

**Figure 4:**
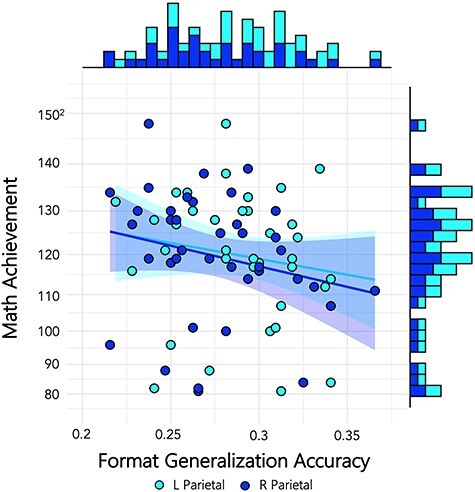
Individual scores for generalization between formats plotted against math achievement. Individual differences in generalization show a negative trending relation to math achievement scores, with effect sizes in line with Bulthé et al. (2018), in both the left parietal (teal) and the right parietal ROI (blue). Dots represent individual classification scores averaged across numerosities.

Our next correlation between classification metrics and mathematics achievement scores closely mirrored the analysis of Bulthé et al. (2018). Bulthé et al. conducted a one-tailed, spearman *rho* correlation and reported a significant negative correlation between math achievement and cross-format generalization accuracy (spearman rho = −0.23, *P* = 0.036, *n* = 63). In Table 3, we report Pearson correlations, which are consistent with our previous analyses (and Lasne et al.), and Spearman correlations, which are consistent with the Bulthé et al. analysis and are less susceptible to the influence of outliers. We also report 2-tailed *P-*values and one-tailed *P-*values in order to compare directly to our previous analyses and the Bulthé et al. analysis. Given Bulthé et al.’s findings, it would be acceptable to hypothesize a negative correlation a priori and specify a one-tailed test, but the effect size of the relation coupled with a Bayes factor is ultimately more informative and thus all information is presented. The current results indicate a small but consistent negative correlation between generalization across number formats in the parietal lobes and math achievement scores that are very similar to the strength of Bulthé et al.’s results. While Bulthé et al. combined the left and right parietal lobe ROIs, we split the ROIs into left and right (Fig. 4). There was a slightly stronger correlation for the right parietal region, where math achievement negatively correlated with generalization accuracy rate [Spearman *rho* (37) = −0.319, one-tailed *P* = 0.024, Kendall’s tau Bayes factor_−0_ = 3.43, indicating moderate support for the negative correlation (3.43 times more likely than the null)]. IFG correlations were not significant and were not accompanied by conclusive evidence for or against the null hypothesis from Bayes factors. Bayes factors for the NFA correlation indicated moderate to strong support for the null. Kendall’s tau Bayes factors were computed in lieu of Spearman *rho* because a Bayesian version of the Spearman tests does not exist in any known software package and Kendall’s tau is an alternative nonparametric test that is robust to the influence of outliers.

**Table 3:** Correlations between nonsymbolic and symbolic generalization accuracy rates and measures of math achievement

		Math achievement		Math achievement
L Parietal	Pearson *r*	−0.172	Spearman *rho*	−0.267^a^
	*P-*value	0.295/0.148	*P-*value	0.100/0.050
	BF_10_^Pr^/BF_−0_^Pr^	0.34/0.57	BF_10_^Kt^/BF_−0_^Kt^	0.94/1.81
R Parietal	Pearson *r*	−0.228	Spearman *rho*	−0.319^a^
	*P-*value	0.163/0.082	*P-*value	0.048/0.024
	BF_10_^Pr^/BF_−0_^Pr^	0.51/0.93	BF_10_^Kt^/BF_−0_^Kt^	1.75/3.43
L IFG	Pearson *r*	−0.164	Spearman *rho*	−0.182
	*P-*value	0.318/0.159	*P-*value	0.269/0.135
	BF_10_^Pr^/BF_−0_^Pr^	0.32/0.54	BF_10_^Kt^/BF_−0_^Kt^	0.41/0.71
R IFG	Pearson *r*	−0.228	Spearman *rho*	−0.190
	*P-*value	0.163/0.082	*P-*value	0.248/0.124
	BF_10_^Pr^/BF_−0_^Pr^	0.51/0.93	BF_10_^Kt^/BF_−0_^Kt^	0.39/0.68
L NFA	Pearson *r*	0.172	Spearman *rho*	0.111
	*P-*value	0.296/0.148	*P-*value	0.502/0.251
	BF_10_^Pr^/BF_−0_^Pr^	0.39/0.10	BF_10_^Kt^/BF_−0_^Kt^	0.24/0.14
R NFA	Pearson *r*	0.008	Spearman *rho*	0.008
	*P-*value	0.959/0.480	*P-*value	0.960/0.480
	BF_10_^Pr^/BF_−0_^Pr^	0.20/0.19	BF_10_^Kt^/BF_−0_^Kt^	0.21/0.20

Notes: ^a^Significant correlation at *P* < 0.05*. P-*values are reported for both 2-tailed and one-tailed tests of correlation. BF_10_ indicates probability of support for a correlation in any direction (similar to 2-tailed test) and BF_−0_ indicates support for the proposed negative correlation (similar to a one-tailed test). ^Pr^Bayes factor for Pearson *r* correlation. ^Kt^Bayes factor for Kendall’s *tau* correlation.

### Cross-Task Generalization and Mathematics Achievement

**Figure 5:**
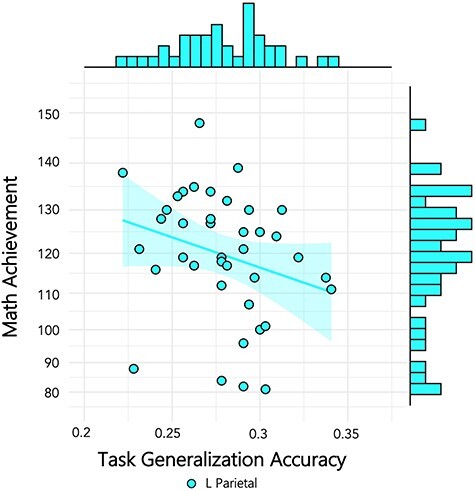
Individual scores for generalization between tasks plotted against math achievement. Individual differences in generalization show a negative trending relation to math achievement scores in the left parietal ROI (teal). Dots represent individual classification scores averaged across numerosities.

Lastly, we investigated whether cross-task generalization (defined as mean task generalization values from Identify to Compare and vice-versa, averaged together) related to mathematics achievement. Analyses and reporting of results follow the same approach as format generalization (Table 4). Results indicate negative correlation between generalization across number formats in the L parietal lobes and math achievement similar to that reported in the cross-format results across both parietal lobes (Spearman *rho* (37) = −0.375, one-tailed *P* = 0.009, Kendall’s tau Bayes factor_−0_ = 5.38, indicating moderate support for the negative correlation [5.38 times more likely than null]) (Fig. 5).

**Table 4:** Correlations between generalization accuracy rates between tasks and measures of math achievement

		Math achievement		Math achievement
L Parietal	Pearson *r*	−0.267^a^	Spearman *rho*	−0.375^a^
	*P-*value	0.100/0.050	*P-*value	0.019/0.009
	BF_10_^Pr^/BF_−0_^Pr^	0.73/1.39	BF_10_^Kt^/BF_−0_^Kt^	2.72/5.38
R Parietal	Pearson *r*	0.099	Spearman *rho*	0.082
	*P-*value	0.548/0.726	*P-*value	0.622/0.689
	BF_10_^Pr^/BF_−0_^Pr^	0.24/0.13	BF_10_^Kt^/BF_−0_^Kt^	0.23/0.15
L IFG	Pearson *r*	0.053	Spearman *rho*	0.056
	*P-*value	0.750/0.625	*P-*value	0.736/0.632
	BF_10_^Pr^/BF_−0_^Pr^	0.21/0.16	BF_10_^Kt^/BF_−0_^Kt^	0.21/0.17
R IFG	Pearson *r*	0.063	Spearman *rho*	0.046
	*P-*value	0.741/0.649	*P-*value	0.786/0.607
	BF_10_^Pr^/BF_−0_^Pr^	0.21/0.15	BF_10_^Kt^/BF_−0_^Kt^	0.22/0.17
L NFA	Pearson *r*	−0.230	Spearman *rho*	−0.261
	*P-*value	0.159/0.080	*P-*value	0.109/0.054
	BF_10_^Pr^/BF_−0_^Pr^	0.52/0.95	BF_10_^Kt^/BF_−0_^Kt^	0.70/1.31
R NFA	Pearson *r*	−0.042	Spearman *rho*	0.070
	*P-*value	0.798/0.399	*P-*value	0.672/0.664
	BF_10_^Pr^/BF_−0_^Pr^	0.21/0.25	BF_10_^Kt^/BF_−0_^Kt^	0.21/0.18

Notes: ^a^Significant correlation at *P* < 0.05*. P*-values are reported for both 2-tailed and one-tailed tests of correlation. BF_10_ indicates probability of support for a correlation in any direction (similar to 2-tailed test) and BF_−0_ indicates support for the proposed negative correlation (similar to a one-tailed test). ^Pr^Bayes factor for Pearson *r* correlation. ^Kt^Bayes factor for Kendall’s *tau* correlation.

### Button Response Check

Both of our tasks require a button press and, as a result, have a significant motor and planning component. In the Identify task, each numerosity required an independent button response. In the Compare task, 2 and 4 shared a button (numbers < 5) while 6 and 8 shared a button (numbers > 5). Motor planning, proprioceptive space, and response selection are known to involve parietal cortex (Simon et al. 2002; Göbel et al. 2004; Grefkes and Fink 2005) and some of the variance in decoding is likely attributable to motor activity. To ensure that numerosity decoding was not simply due to non-numerical, motor, and motor-planning neural activity in the most likely ROI to suffer this confound, we compared confusion rates (i.e., prediction rates when the classifier is incorrect) between numerosities that shared buttons, and those that did not, to check for a bias according to button press in both the left and right parietal ROIs in the Compare task collapsed across formats. In these conditions we can compare variance in models predicted by distance to variance predicted by button response. Since numerosities 2 and 4 share a button, then a classifier capturing neural activity associated with button response rather than number would confuse 2 and 4, but not 6 and 8. On the other hand, numerosity encoding is also expected to follow a confusion distribution based on the distance effect, where 4 is equally likely to be confused with 2 and 6 (distance = 2), but not with 8 (distance = 4) (Bulthé et al. 2014; Bulthé et al. 2015). It should be stated that these analyses were completed posthoc and were not included as part of the original preregistration of analyses.

**Figure 6:**
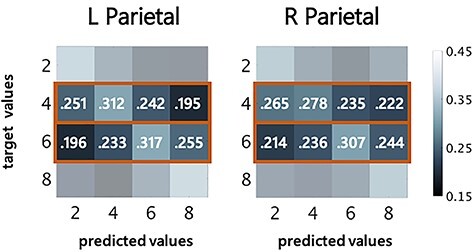
Average classification predictions for target values 4 and 6 in left and right parietal ROI during comparison task across participants. The comparison task shares only some button response values but demonstrates a linear distance effect, indicating that classification was likely capturing numerical information in activation patterns. Confusion matrices are the same as those presented in Figure 2 for the comparison task, but numerical values are detailed here for variables that were of interest for the “button response check”. Boxes in bold orange outline indicate target values that were included as variables in the tests for a linear effect of distance on accuracy rate; see also Supplementary Tables S21 and S22. Color bar represents classification rate as a percentage.

In the left parietal ROI, when 4 was the presented numerosity, on average, the classifier predicted numerosity 2 at a rate of 25.1% and 6 at a rate of 24.2%, which did not differ significantly [*t*(38) = 0.54, *P* = 0.593] (Fig. 6, left). In the right parietal ROI, when 4 was the presented numerosity, the classifier predicted numerosity 2 at an accuracy rate of 26.5% and 6 at a rate of 23.5%, which did not differ significantly [*t*(38) = 1.89, *P* = 0.067] (Fig. 6, right). When 6 was the numerosity seen by a participant, in the left parietal ROI the classifier predicted numerosity 4 at an accuracy rate of 23.3% and 8 at a rate of 25.5%, which did not differ significantly [*t*(38) = −1.33, *P* = 0.191].

To explore the linear effect of distance on accuracy rate, prediction rate was run as a mixed model, one model for the left parietal ROI and one for the right parietal ROI, predicting rate of classifier prediction from the numerical distance from 4 and 6 (e.g., distance of 2 from 4 = 2, distance of 4 from 4 = 0, distance of 6 from 4 = 2, distance of 8 from 4 = 4), where the intercepts and slopes of participants were allowed to vary randomly in the model to account for the within-subject nature of the data (for further model details, see Supplementary Tables S21 and S22). In the left parietal lobe, distance was significant predictor of confusion rate [*t*(38) = −7.30, *P* < 0.001], but button response was not [*t*(38) = 0.57, *P* = 0.573]. In the right parietal lobe, again distance was a significant predictor of confusion rate [*t*(38) = −4.64, *P* < 0.001], but button response was not [*t*(38) = 0.488, *P* = 0.628].

The pattern of results in both the *t*-tests above and mixed linear models exploring the distance effect indicate that neural patterns of activation were successfully capturing information about numerosity and were not significantly influenced by button response.

## Discussion

The current study addressed 3 questions. First, does number representation share cortical patterns of activation across formats (Nonsymbolic versus Symbolic)? To investigate, we assessed whether multivariate patterns of neural response to specific numerical magnitudes in one format can generalize to the other using MVPA at 7 T fMRI. Second, are representations of number task-dependent? Again, we assessed whether neural activation patterns can generalize across the Compare and Identify tasks. Third, do patterns of neural response to number (i.e., decoding performance and generalization across formats and tasks) correlate with behavioral metrics of numerical ability measured by (a) out-of-scanner number comparison tasks and (b) math achievement.

### Decoding

We first established that decoding of numerosities within task and within format was successful in 7 of the 8 ROIs, including the bilateral parietal lobes, bilateral IFG, left NFA, and bilateral parietal + NFA (a combination of both ROIs within-hemisphere), excluding only the right NFA. Classification accuracy in both left and right NFA regions was the lowest of all ROIs for all conditions. Given the field’s newly emerging understanding of the role of the NFA (Yeo et al. 2017), we tested the hypothesis that patterns of activation in the parietal lobe combined with the NFA might provide higher rates of discriminability than either region alone. This hypothesis can be rejected. Though decoding in the parietal + NFA ROI was higher than the NFA region alone, it was not higher than the parietal region alone, indicating that information from the parietal lobe was driving decoding performance in the parietal + NFA ROI. Lower decoding performance of the parietal + NFA ROI than the parietal ROI alone is likely due to the loss of informative voxels from the parietal lobe when the 2 regions were combined. In order to maintain ROIs of 600 voxels, the most active 300 voxels from each ROI (based on the all conditions vs. baseline contrast) were selected to ensure equal representation across ROIs. Of note, however, is that we found evidence of successful decoding within the left NFA even for Nonsymbolic numerosities, indicating that the left NFA may have some role beyond symbol recognition. Recent work has found evidence a problem-size effect in this region (Pinheiro-Chagas et al. 2018) as well as a preference for mathematical processing beyond the involvement of numerals (Grotheer et al. 2018). This activity may indicate a role beyond simple visual form recognition.

Both of the tasks used in the current study are active tasks that require a button response with different fingers for each numerosity, which cannot be isolated from neural activity associated with processing numerical information in the current study design. As such, this influences the interpretation of all findings of the current study. Both decoding and generalization results should be interpreted as involving mechanisms beyond simply perception of number, but also decisional processes related to identification and comparison. In other words, evidence of shared neural resources for numerosity-specific processing across formats or tasks should be interpreted to include more active processing of those numerosities than a delayed comparison task where neural activation is being modeled during the perception of the first number.

To explore whether button press or numerosity was driving classification accuracy, we analyzed activity in portions of our experiment where button response and numerosity could be dissociated. Results indicated that decoding accuracy rates were driven by processing of numerical information and not button-response selection (see Supplementary Tables S21 and S22). However, button response does not capture all active components of the tasks beyond the processing of number. For example, it is conceivable that attentional mechanisms are engaged to a different degree across numerical stimuli, varying collinearly with numerical distance in the number comparison task. In this case, showing that the distance effect drives our results does not completely mitigate concerns that decoding is capturing, for example, attentional mechanisms related to numerical information.

### Generalization

In the current study, the LDA classifier was able to train on Nonsymbolic numerosities and predict the numerosities of Symbolic stimuli at above-chance accuracy rates, and vice versa, in the bilateral parietal lobes. These findings are in agreement with some previous studies that have found evidence for between-format generalization (Eger et al. 2009; Damarla and Just 2013; Teichmann et al. 2018; Bankson et al. 2019) but in disagreement with others (Bulthé et al. 2014, 2015).

As mentioned, the current study most closely resembles Eger et al. (2009) and Bulthé et al. (2014) based on both stimuli and task design, which each come to different conclusions regarding shared patterns of activation between formats. The current study, Eger et al. (2009), and Bulthé et al. (2014), all use the numbers 2, 4, 6, and 8 represented as dots and digits. However, there are also several key differences. First, the current study more than doubles the sample size of the other 2 studies (Bulthé et al. 2014*n* = 16; Eger et al. 2009*n* = 10; current study *n* = 39). Second, the current study used 7 T ultra-high field fMRI, which increases the signal-to-noise ratio of the BOLD response (Yacoub et al. 2001; van der Zwaag et al. 2009; De Martino et al. 2011, 2008). Third, Bulthé et al. included more trials (72–84 trials), but their short-block fMRI design diverges significantly from a typical event-related or block design in that many exemplars are spaced only 800 ms from other exemplars, possibly reducing separability of the estimated BOLD response for each condition. We ran a typical event-related design with an average ISI of 5.3 s. Eger et al. included 32 trials per condition. In the current analysis, collapsing across task (when decoding within format) or format (when decoding within task), there were 40 trials per condition. All of these differences led to increased power in the current study to detect the presence of generalization, which may be one reason that it differed with the results presented in Bulthé et al. (2014). However, it should be noted that generalization across formats was still observed when the tasks were analyzed separately with fewer exemplars (20 per condition).

Another difference between the current study and most previous analyses is that we used an LDA classifier. Prior to running analyses with numbers as conditions, we compared the SVM and LDA classifiers implemented in the CoSMo MVPA toolbox in the motor cortex with button responses as conditions of interest as a data quality and data processing check (for detailed comparisons, see Gokcen and Peng 2002; Mandelkow et al. 2016; Misaki et al. 2010). The LDA classifier consistently outperformed the SVM classifier, and so we decided to use the LDA classifier for the main analysis. Both Eger et al. and Bulthé et al. use SVM classifiers in their analysis, which could also lead to differences in the findings.

A further contribution of the current study is that classification generalized successfully across the Identify and Compare tasks in bilateral parietal and IFG regions. This indicates that number-specific activation patterns are shared in all 4 of these regions across tasks. Simply identifying the numbers as a 2, 4, 6, or 8 is enough to activate representations similar to those elicited in a comparison task, and importantly, these data suggest the representation of 2, or 4, or 6, or 8, is the same representation whether you are processing the magnitude or simply identifying it. The fact that the Identify and Compare tasks used different button responses makes this finding unlikely to be driven by motor or response selection demands and more likely to be driven by semantic similarity.

Still, as with all fMRI, each functional voxel includes hundreds of thousands of neurons. Therefore, it may be that the functional resolution of MRI does not accurately capture independent populations of neurons within a voxel that are each dedicated only to a specific format. If these independent populations existed for each format or task, and were close enough to each other and laid out in the same numerosity-specific pattern across the cortex, their independent BOLD response could appear the same at the level of a functional MRI voxel. Further fine-grained analysis at the level of neural circuits is likely necessary to make conclusions directly related to actual neural recycling (Dehaene and Cohen 2007).

### Classification–Behavior Correlations

We also tested 3 correlations that used classification rates as individual differences metrics to predict number comparison performance and math achievement.

The first set of classification–behavior correlations centered on the idea that decoding accuracy within a given format may provide a metric of the acuity of numerical representation that would correlate with behavioral performance in an out-of-scanner number comparison task. If individuals with greater numerical acuity have sharper tuning curves that are more distinct, it could follow that discriminability in the context of a multivoxel analysis would also be greater, and in turn, that their behavioral performance should be better. This method has been used successfully to relate behaviors of phoneme detection discriminability to MVPA phoneme decoding (Raizada et al. 2010) and previously in relation to numerosity discrimination. Although there are substantial methodological differences from the current study, Lasne et al. (2018) reported that decoding accuracy of numerosities in the right parietal lobe of a nonsymbolic number comparison task correlated with behavioral Weber fractions in an independent number comparison task with an effect size of *r* = −0.59. This correlation increased to *r* = −0.74 when they isolated the effect to the homologue of the right lateral intraparietal region of macaques compared with the left and ventral parietal regions, which showed lower rates of correlation.

In contrast, the current results showed no correlation between decoding accuracy and behavioral performance across any of the ROIs. We first used a performance score as planned, which is a response time metric adjusted for accuracy, because this metric is better suited to the high accuracy rates associated with symbolic number comparison, which was also a planned analysis. However, after finding no significant correlation, we also computed Weber fractions to more closely match the analysis of Lasne et al., which again provided no evidence for a correlation in the right parietal ROI [*r* = 0.093, BF_01_ = 4.31]. In fact, the Bayes factor indicated moderate support for the null. Several differences exist between the 2 studies that may have led to a difference in results. First, the behavioral and fMRI delayed numerosity comparison tasks in the Lasne et al. study were more closely matched than in the current study, which could have led to a higher correlation. For example, in the current study, the numerosities were 2, 4, 6, and 8 in the scanner (compared to a constant, i.e., 5) created based on the Dehaene method for generating dot stimuli (Dehaene et al. 2005) but included a wider range of numerosities in the behavioral comparison task (i.e., 5–15 for nonsymbolic, 2–9 for symbolic) created using the Gebuis method (Gebuis and Reynvoet 2011). Lasne et al. used the same numerosities (8–34) both inside and outside of the scanner and used the same stimuli generation method for each. Also, it should also be noted the Lasne et al. numbers are all considered outside of subitizing range, whereas the current study’s numerosities spanned the subitizing range and beyond for the in-scanner task. Secondly, Lasne et al.’s sample reported very high acuity with a small range of ability [mean *w* = 0.15; range = 0.13–0.19] compared to the current sample [mean *w* = 0.23, range = 0.09 to 0.34]. Task variations may greatly influence estimations of Weber fractions, but a massive online study of the Panamath task estimates a mean *w* for a sample of young adult participants to be about 0.25 (Halberda et al. 2012), suggesting that our sample was about average. In comparison, Lasne et al.’s sample had exceptional acuity. Third, the method for calculating weber fractions differed between the 2 studies. Different methods of calculating weber fractions lead to different distributions, so the weber fractions are not directly comparable. Fourth, the current study modeled neural response to number in the context of 2 active tasks, but Lasne et al. decoded numerosities during the perception portions of the task, which minimized other task-active cognitive processes, such as response selection. Lastly, it should be noted that the current sample is much larger at *n* = 39 compared to Lasne et al.’s *n* = 12. Brain–behavior correlations in small samples may increase the chances for a false positive or overestimation of the effect size (Cremers et al. 2017). Replication of both findings with a larger sample size and broader range of abilities will be necessary for resolution of this issue.

The second set of classification–behavior correlations tested whether decoding performance correlated with math achievement rates in the current sample. Our results demonstrated that decoding accuracy did not correlate with math achievement in either format across any of the ROIs in the current study. Given that our decoding accuracies did not correlate with an independent metric of behavioral numerical acuity, these results suggest either that MVPA decoding accuracy in the current study context does not index the acuity of numerical representation precision, or that such representational acuity is not what drives the observed links between performance on out-of-scanner number comparison tasks and math competence. To check how our behavioral number comparison tasks related to math achievement and its subtests, we also ran these correlations (see Supplementary Table 23). Results showed only the symbolic performance metric correlated with math fluency. So, the lack of a correlation between decoding and math achievement could be due to the fact that the current study’s indices of numerical acuity as measured by symbolic and nonsymbolic number comparison tasks are less correlated with mathematics achievement in the current sample than other studies using similar tasks.

The final set of classification–behavior correlations tested if generalization between formats and tasks related to math achievement. Based on the idea that representations of symbolic and nonsymbolic number become increasingly specialized over development, a divergence in neural patterns between symbolic and nonsymbolic formats may relate to more developed numerical abilities associated with math achievement. Bulthé et al. (2018) reported evidence in favor of this hypothesis, showing a negative correlation between generalization rate across numerical format in the bilateral parietal lobes and arithmetic skills with an effect size of Spearman *rho =* −0.23 (*n* = 63). Based on this finding, we would expect, a priori, to see similar results in the parietal lobes. However, we also expanded the search by including the IFG and NFA and by splitting regions into left and right hemispheres. Results converged with those of Bulthé et al., whereby generalization between numerical formats negatively correlates with math achievement, most highly in the right parietal ROI [Spearman *rho* = −0.319, one-tailed *P* = 0.024, BF_−0_ = 3.43]. The correlation is slightly lower in the left parietal ROI but trending in the same direction. Considering how closely the current results fit with those of Bulthé et al., these results lend further support to the idea that lower cross-format generalization rates are capturing a divergence or “estrangement” (Lyons et al. 2012) in patterns of neural activity between formats that is associated with greater math skills. Results for the task generalization and math achievement correlation indicated a similar negative correlation in the left parietal ROI. On average, individuals with worse generalization of numerosity-specific activation patterns between the Identify and Compare tasks had higher math scores (Spearman *rho* = −0.375, one-tailed *P* = 0.009, BF_−0_ = 5.38). Or, in other words, more task-specific numerosity representations were associated with higher math scores. This could be an independent effect from format generalization, whereby representational specificity is indexed specific to the task. More proficient mathematical thinkers could elicit more task-specific engagement in the contexts of identifying numbers as nominative objects versus comparing numbers in a computational context. However, taken together with the format-generalization finding, these negative correlations could indicate a broader trend than either the decoupling between formats or task-specific engagement hypotheses. They could point towards a more general increase in specialization for cognitive processes related to numerical processing associated with mathematical proficiency. Still, this novel finding should be further replicated and investigated across multiple age groups in order to understand how specialization may unfold over development.

## Conclusion

The current study set out to address whether patterns of neural activity associated with processing numerosities is shared across formats and tasks, and further, if those patterns relate to individual differences in number comparison behaviors and math achievement. We successfully trained a classifier to discriminate between numerosities represented as dots and generalize at above-chance accuracy rates to the same numerosities represented as Arabic digits, and vice versa, in the bilateral parietal lobe and to some extent, the left IFG. This indicates that at some level, numerosity-specific neural resources are shared between formats, and further, that both the left and right parietal lobes are directly involved in the encoding of numerosity to the extent that numerosity-specific decoding was successful within each hemisphere independently. Generalization was also successful across tasks where participants either identified numbers or compared them, suggesting task-independent shared neural resources in the bilateral parietal lobes and bilateral IFG. While a significant amount of evidence points to the involvement of the dorsolateral prefrontal cortex as being involved with number processing (Sokolowski et al. 2016; Arsalidou et al. 2017; Zhang et al. 2018), the current results indicate that this processing is specific to individual numbers in multiple formats and task contexts. Lastly, in correlating our decoding and generalization metrics with independent behavioral measures, we found that decoding performance did not relate to number comparison performance outside of the scanner or math ability, but generalization between formats and between tasks in the parietal lobes did negatively relate to math achievement. Together, these findings suggest that individual differences in representational specificity within format and task contexts relates to mathematical expertise.

## Supplementary Material

FormatTaskandAcuity_7T_SUPPLEMENT_CerebralCortexCommunications_R1_tgaa038
