# Supplementary material for: Shared Numerosity Representations Across Formats and Tasks Revealed with 7 Tesla fMRI: Decoding, Generalization, and Individual Differences in Behavior
Source: Cereb Cortex Commun. 2020 Jul 30;1(1):tgaa038. doi: 10.1093/texcom/tgaa038 (PMC8153058; doi:10.1093/texcom/tgaa038)
Supplement: FormatTaskandAcuity_7T_SUPPLEMENT_CerebralCortexCommunications_R1_tgaa038 [file formattaskandacuity_7t_supplement_cerebralcortexcommunications_r1_tgaa038.docx]

SUPPLEMENTARY MATERIALS

**
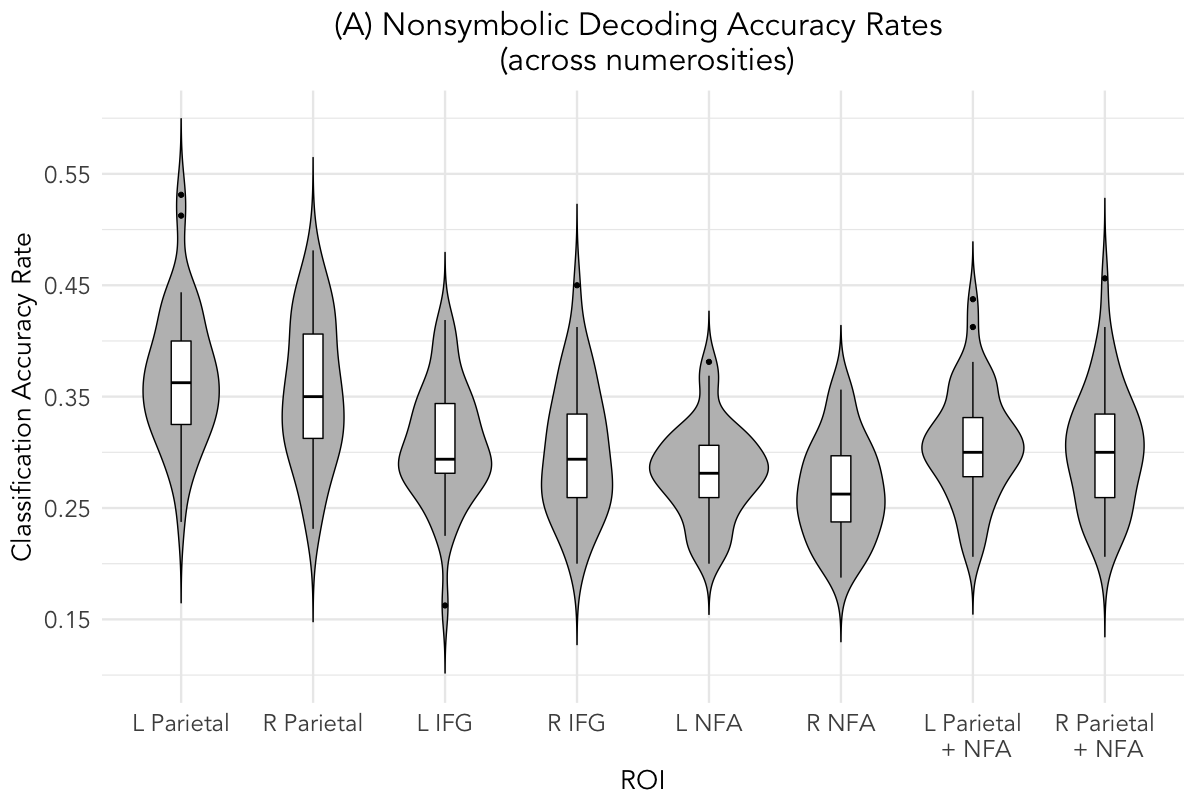
**

**
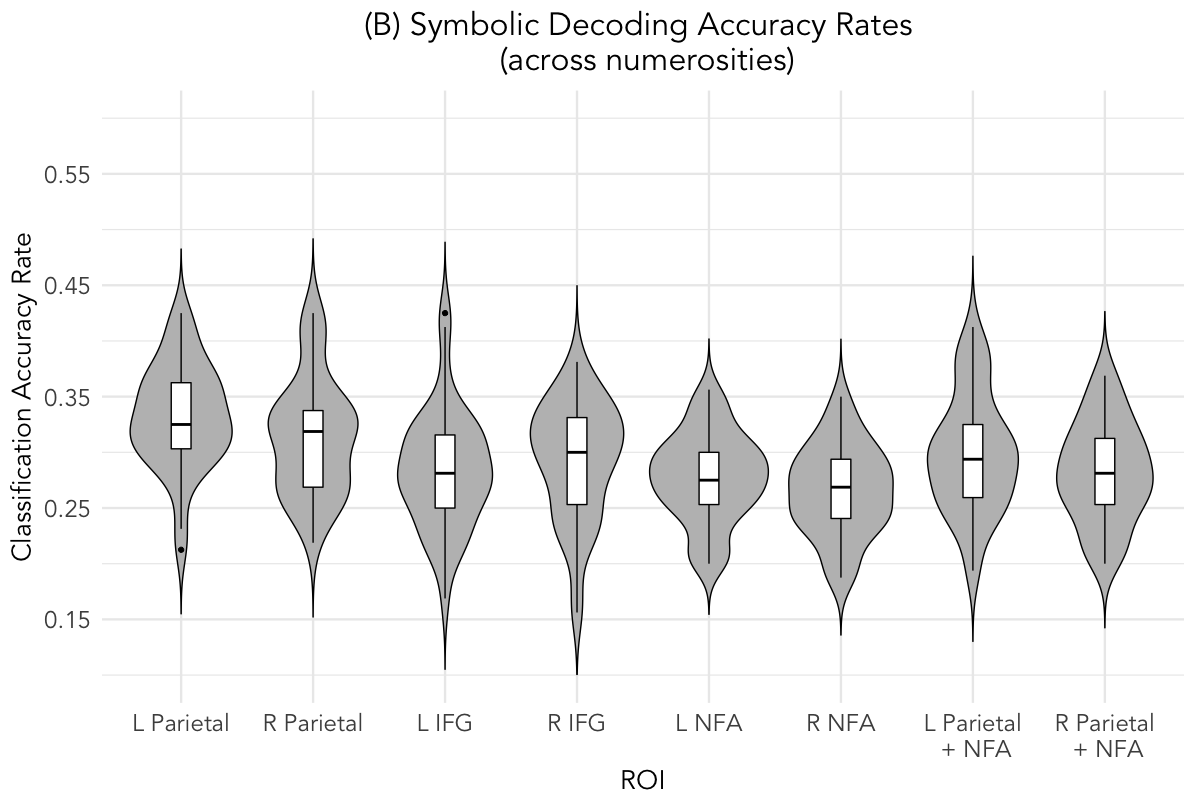
**

**Figure S1. Distribution of numerosity decoding within the (A) nonsymbolic and (B) symbolic format.** Classification Accuracy Rate = average classification across numerosities within ROI; L = Left; R = Right; IFG = inferior frontal gyrus; NFA = number form area. Box plot hinges represent 25th and 75th percentile of distributions, whiskers extend from hinge to the largest value not beyond 1.5 times the interquartile range. All points plotted beyond whiskers.

**
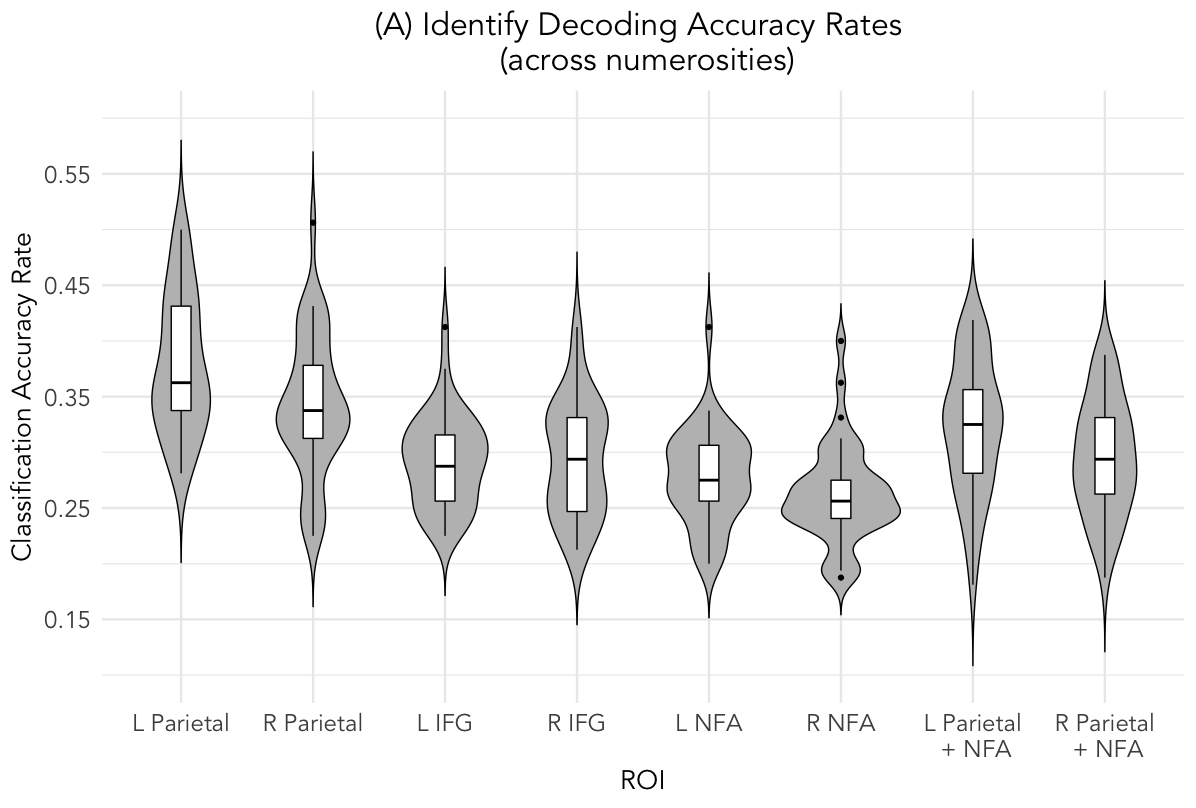
**

**
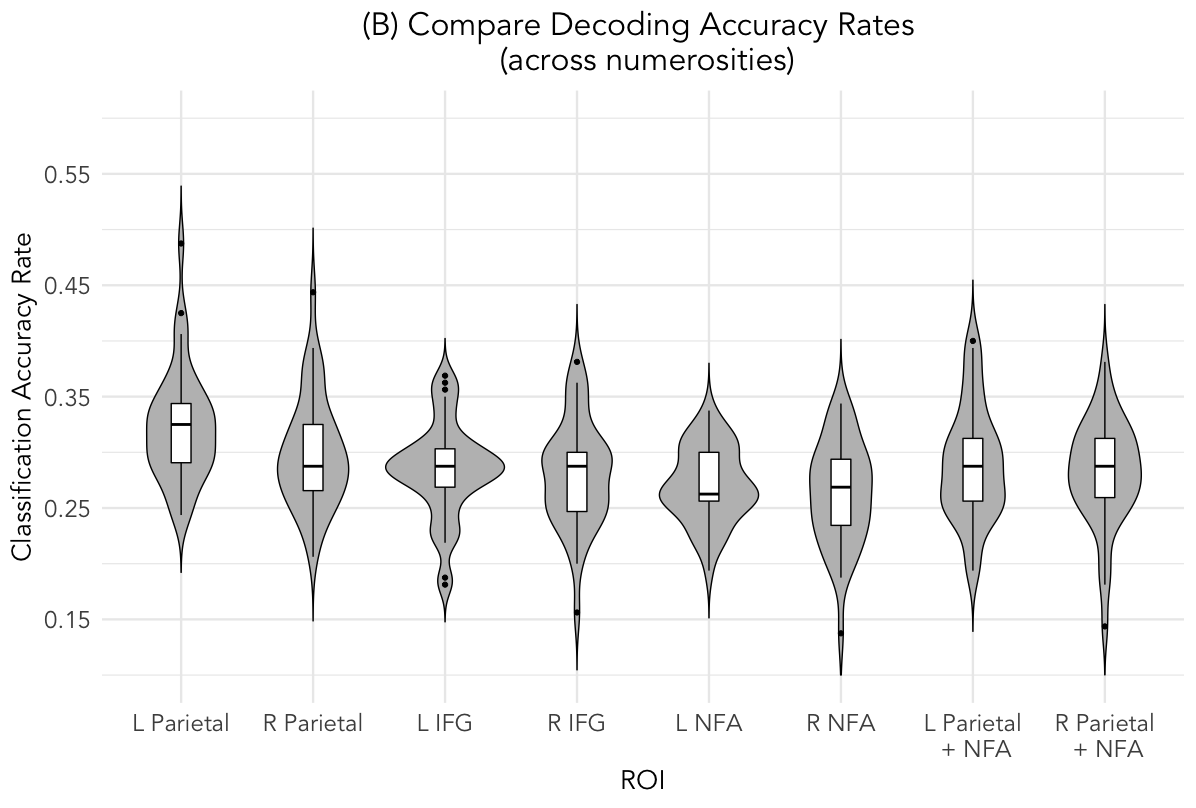
**

**Figure S2. Distribution of numerosity decoding within the (A) identify task and the (B) compare task.** Classification Accuracy Rate = average classification across numerosities within ROI; L = Left; R = Right; IFG = inferior frontal gyrus; NFA = number form area. Box plot hinges represent 25th and 75th percentile of distributions, whiskers extend from hinge to the largest value not beyond 1.5 times the interquartile range. All points plotted beyond whiskers.

**Supplementary Table S1.** Details for symbolic and nonsymbolic number comparison tasks performed outside of the MRI scanner during a separate testing session.

| Symbolic Number Comparison | | | |
| --- | --- | --- | --- |
| Ratios | Stimuli | Accuracy | RT |
| 0.33 | 2 v 6 | 0.995 | 460 |
| 0.50 | 4 v 8 | 0.995 | 467 |
| 0.67 | 2 v 3 | 0.990 | 478 |
| 0.80 | 4 v 5 | 0.985 | 499 |
| 0.86 | 6 v 7 | 0.944 | 558 |
| 0.88 | 7 v 8 | 0.941 | 546 |
| 0.90 | 8 v 9 | 0.979 | 545 |
| Nonsymbolic Number Comparison | | | |
| 0.33 | 5 v 15 | 0.997 | 542 |
| 0.50 | 5 v 10 | 0.992 | 612 |
| 0.67 | 6 v 9 | 0.982 | 653 |
| 0.80 | 8 v 10 | 0.772 | 782 |
| 0.86 | 12 v 14 | 0.649 | 822 |
| 0.88 | 7 v 8 | 0.572 | 925 |
| 0.89 | 9 v 10 | 0.521 | 901 |

| **Supplementary Table S2.** Decoding Accuracy for Symbolic Numerosities: Identify. | | | | | | | | | | | | | | | | | |
| --- | --- | --- | --- | --- | --- | --- | --- | --- | --- | --- | --- | --- | --- | --- | --- | --- | --- |
|  |  |  |  |  |  |  |  |  |  |  |  |  |  |  |  |  |  |
|  | | **L Par** | | **R Par** | | **L IFG** | | **R IFG** | | **L NFA** | | **R NFA** | | **L Par + NFA** | | **R Par + NFA** | |
| Mean |  | 0.338 |  | 0.301 |  | 0.273 |  | 0.278 |  | 0.281 |  | 0.263 |  | 0.334 |  | 0.317 |  |
| SD |  | 0.0703 |  | 0.0730 |  | 0.0573 |  | 0.0623 |  | 0.0529 |  | 0.0578 |  | 0.0695 |  | 0.0763 |  |
| Minimum |  | 0.212 |  | 0.138 |  | 0.188 |  | 0.150 |  | 0.200 |  | 0.163 |  | 0.237 |  | 0.175 |  |
| Maximum |  | 0.512 |  | 0.450 |  | 0.400 |  | 0.412 |  | 0.388 |  | 0.425 |  | 0.500 |  | 0.463 |  |
|  | | | | | | | | | | | | | | | | | |

| **Supplementary Table S3.** Decoding Accuracy for Symbolic Numerosities: Compare. | | | | | | | | | | | | | | | | | |
| --- | --- | --- | --- | --- | --- | --- | --- | --- | --- | --- | --- | --- | --- | --- | --- | --- | --- |
|  |  |  |  |  |  |  |  |  |  |  |  |  |  |  |  |  |  |
|  | | **L Par** | | **R Par** | | **L IFG** | | **R IFG** | | **L NFA** | | **R NFA** | | **L Par + NFA** | | **R Par + NFA** | |
| Mean |  | 0.313 |  | 0.292 |  | 0.291 |  | 0.292 |  | 0.275 |  | 0.276 |  | 0.285 |  | 0.282 |  |
| SD |  | 0.0498 |  | 0.0590 |  | 0.0540 |  | 0.0540 |  | 0.0507 |  | 0.0550 |  | 0.0630 |  | 0.0574 |  |
| Minimum |  | 0.163 |  | 0.150 |  | 0.0875 |  | 0.188 |  | 0.188 |  | 0.163 |  | 0.163 |  | 0.163 |  |
| Maximum |  | 0.400 |  | 0.400 |  | 0.375 |  | 0.400 |  | 0.425 |  | 0.388 |  | 0.425 |  | 0.400 |  |
|  | | | | | | | | | | | | | | | | | |

| **Supplementary Table S4.** Decoding Accuracy for Nonsymbolic Numerosities: Identify. | | | | | | | | | | | | | | | | | |
| --- | --- | --- | --- | --- | --- | --- | --- | --- | --- | --- | --- | --- | --- | --- | --- | --- | --- |
|  |  |  |  |  |  |  |  |  |  |  |  |  |  |  |  |  |  |
|  | | **L Par** | | **R Par** | | **L IFG** | | **R IFG** | | **L NFA** | | **R NFA** | | **L Par + NFA** | | **R Par + NFA** | |
| Mean |  | 0.393 |  | 0.381 |  | 0.311 |  | 0.320 |  | 0.274 |  | 0.267 |  | 0.386 |  | 0.371 |  |
| SD |  | 0.0984 |  | 0.0910 |  | 0.0770 |  | 0.0769 |  | 0.0622 |  | 0.0575 |  | 0.0995 |  | 0.0700 |  |
| Minimum |  | 0.200 |  | 0.188 |  | 0.163 |  | 0.175 |  | 0.163 |  | 0.163 |  | 0.175 |  | 0.237 |  |
| Maximum |  | 0.637 |  | 0.662 |  | 0.463 |  | 0.625 |  | 0.425 |  | 0.388 |  | 0.588 |  | 0.563 |  |
|  | | | | | | | | | | | | | | | | | |

| **Supplementary Table S5.** Decoding Accuracy for Nonsymbolic Numerosities: Compare. | | | | | | | | | | | | | | | | | |
| --- | --- | --- | --- | --- | --- | --- | --- | --- | --- | --- | --- | --- | --- | --- | --- | --- | --- |
|  |  |  |  |  |  |  |  |  |  |  |  |  |  |  |  |  |  |
|  | | **L Par** | | **R Par** | | **L IFG** | | **R IFG** | | **L NFA** | | **R NFA** | | **L Par + NFA** | | **R Par + NFA** | |
| Mean |  | 0.312 |  | 0.309 |  | 0.279 |  | 0.281 |  | 0.232 |  | 0.265 |  | 0.282 |  | 0.273 |  |
| SD |  | 0.0671 |  | 0.0730 |  | 0.0587 |  | 0.0649 |  | 0.0604 |  | 0.0689 |  | 0.0689 |  | 0.0638 |  |
| Minimum |  | 0.163 |  | 0.188 |  | 0.138 |  | 0.163 |  | 0.113 |  | 0.100 |  | 0.163 |  | 0.175 |  |
| Maximum |  | 0.450 |  | 0.450 |  | 0.412 |  | 0.425 |  | 0.350 |  | 0.400 |  | 0.463 |  | 0.450 |  |

| **Supplementary Table S6.** Generalization Accuracy for Nonsymbolic to Symbolic | | | | | | | | | | | | | | | | | | |
| --- | --- | --- | --- | --- | --- | --- | --- | --- | --- | --- | --- | --- | --- | --- | --- | --- | --- | --- |
|  |  |  | |  |  |  |  |  |  |  |  |  |  |  |  |  |  |  |
|  | | **L Par** | | | **R Par** | | **L IFG** | | **R IFG** | | **L NFA** | | **R NFA** | | **L Par + NFA** | | **R Par + NFA** | |
| *t* | | 5.52 | |  | 3.48 |  | 2.47 |  | 0.81 |  | 1.06 |  | -0.28 |  | 4.23 |  | 1.45 |  |
| *p* | | | <.001 |  | .010 |  | .146 |  | 1.000 |  | 1.000 |  | 1.000 |  | .001 |  | 1.000 |  |
| Mean |  | 0.288 | |  | 0.271 |  | 0.263 |  | 0.255 |  | 0.256 |  | 0.249 |  | 0.276 |  | 0.258 |  |
| SD |  | 0.0433 | |  | 0.0374 |  | 0.0328 |  | 0.0369 |  | 0.0329 |  | 0.0326 |  | 0.0386 |  | 0.0332 |  |
| *Note*. p-values are Bonferroni adjusted for testing across 8 ROIs | | | | | | | | | | | | | | | | | | |

| **Supplementary Table S7.** Generalization Accuracy for Symbolic to Nonsymbolic | | | | | | | | | | | | | | | | | |
| --- | --- | --- | --- | --- | --- | --- | --- | --- | --- | --- | --- | --- | --- | --- | --- | --- | --- |
|  |  |  |  |  |  |  |  |  |  |  |  |  |  |  |  |  |  |
|  | | **L Par** | | **R Par** | | **L IFG** | | **R IFG** | | **L NFA** | | **R NFA** | | **L Par + NFA** | | **R Par +**  **NFA** | |
| *T* |  | 7.12 |  | 3.85 |  | 3.11 |  | 1.42 |  | 0.16 |  | -0.03 |  | 3.26 |  | 0.54 |  |
| *P* | | < .001 |  | .004 |  | .028 |  | 1.000 |  | 1.000 |  | 1.000 |  | .019 |  | 1.000 |  |
| Mean |  | 0.294 |  | 0.279 |  | 0.262 |  | 0.258 |  | 0.251 |  | 0.250 |  | 0.720 |  | 0.253 |  |
| SD |  | 0.0385 |  | 0.0476 |  | 0.0251 |  | 0.0374 |  | 0.0317 |  | 0.0375 |  | 0.0378 |  | 0.0353 |  |
| *Note*. p-values are Bonferroni adjusted for testing across 8 ROIs | | | | | | | | | | | | | | | | | |

| **Supplementary Table S8.** Generalization Accuracy for Identify: Nonsymbolic to Symbolic | | | | | | | | | | | | | | | | | |
| --- | --- | --- | --- | --- | --- | --- | --- | --- | --- | --- | --- | --- | --- | --- | --- | --- | --- |
|  |  |  |  |  |  |  |  |  |  |  |  |  |  |  |  |  |  |
|  | | **L Par** | | **R Par** | | **L IFG** | | **R IFG** | | **L NFA** | | **R NFA** | | **L Par + NFA** | | **R Par + NFA** | |
| Mean | | 0.306 |  | 0.276 |  | 0.264 |  | 0.258 |  | 0.254 |  | 0.231 |  | 0.276 |  | 0.263 |  |
| SD | | 0.0590 |  | 0.0583 |  | 0.0532 |  | 0.0569 |  | 0.0464 |  | 0.0517 |  | 0.0506 |  | 0.0561 |  |
| *t* | | 5.90 |  | 2.78 |  | 1.66 |  | 0.91 |  | 0.52 |  | -2.25 |  | 3.16 |  | 1.46 |  |
| *p_bonferroni_* | | < .001 |  | 0.067 |  | 0.0849 |  | 1.000 |  | 1.000 |  | .245 |  | .025 |  | 1.000 |  |

| **Supplementary Table S9.** Generalization Accuracy for Identify: Symbolic to Nonsymbolic | | | | | | | | | | | | | | | | | |
| --- | --- | --- | --- | --- | --- | --- | --- | --- | --- | --- | --- | --- | --- | --- | --- | --- | --- |
|  |  |  |  |  |  |  |  |  |  |  |  |  |  |  |  |  |  |
|  | | **L Par** | | **R Par** | | **L IFG** | | **R IFG** | | **L NFA** | | **R NFA** | | **L Par + NFA** | | **R Par + NFA** | |
| Mean | | 0.317 |  | 0.289 |  | 0.255 |  | 0.255 |  | 0.255 |  | 0.254 |  | 0.267 |  | 0.261 |  |
| SD | | 0.0672 |  | 0.0577 |  | 0.0387 |  | 0.0501 |  | 0.0492 |  | 0.0453 |  | 0.0557 |  | 0.0458 |  |
| *t* | | 6.19 |  | 4.26 |  | 0.83 |  | 0.60 |  | 0.57 |  | 0.49 |  | 1.94 |  | 1.49 |  |
| *p_bonferroni_* | | < .001 |  | .001 |  | 1.000 |  | 1.000 |  | 1.000 |  | 1.000 |  | .476 |  | 1.000 |  |

| **Supplementary Table S10.** Generalization Accuracy for Compare: Nonsymbolic to Symbolic | | | | | | | | | | | | | | | | | |
| --- | --- | --- | --- | --- | --- | --- | --- | --- | --- | --- | --- | --- | --- | --- | --- | --- | --- |
|  |  |  |  |  |  |  |  |  |  |  |  |  |  |  |  |  |  |
|  | | **L Par** | | **R Par** | | **L IFG** | | **R IFG** | | **L NFA** | | **R NFA** | | **L Par + NFA** | | **R Par + NFA** | |
| Mean | | 0.278 |  | 0.251 |  | 0.256 |  | 0.251 |  | 0.261 |  | 0.251 |  | 0.258 |  | 0.258 |  |
| SD | | 0.0564 |  | 0.0588 |  | 0.0479 |  | 0.0441 |  | 0.0474 |  | 0.0490 |  | 0.0498 |  | 0.0538 |  |
| *t* | | 3.05 |  | 0.14 |  | 0.84 |  | 0.18 |  | 0.17 |  | 1.35 |  | 1.00 |  | 0.97 |  |
| *p_bonferroni_* | | .033 |  | 1.000 |  | 1.000 |  | 1.000 |  | 1.000 |  | 1.000 |  | 1.000 |  | 1.000 |  |

| **Supplementary Table S11.** Generalization Accuracy for Compare: Symbolic to Nonsymbolic | | | | | | | | | | | | | | | | | |
| --- | --- | --- | --- | --- | --- | --- | --- | --- | --- | --- | --- | --- | --- | --- | --- | --- | --- |
|  |  |  |  |  |  |  |  |  |  |  |  |  |  |  |  |  |  |
|  | | **L Par** | | **R Par** | | **L IFG** | | **R IFG** | | **L NFA** | | **R NFA** | | **L Par + NFA** | | **R Par + NFA** | |
| Mean | | 0.287 |  | 0.258 |  | 0.254 |  | 0.256 |  | 0.256 |  | 0.249 |  | 0.265 |  | 0.267 |  |
| SD | | 0.0479 |  | 0.0542 |  | 0.0360 |  | 0.0495 |  | 0.0445 |  | 0.0516 |  | 0.0431 |  | 0.0501 |  |
| *t* | | 4.85 |  | 0.89 |  | 0.78 |  | 0.81 |  | 0.81 |  | -0.12 |  | 2.23 |  | 0.88 |  |
| *p_bonferroni_* | | <.001 |  | 1.000 |  | 1.000 |  | 1.000 |  | 1.000 |  | 1.000 |  | .253 |  | 1.000 |  |

| **Supplementary Table S12.** Format generalization accuracy across all analyses. Numbers refer to t-statistic values from the comparison of mean classification across numerosities compared to chance (25%). | | | | | | | | |
| --- | --- | --- | --- | --- | --- | --- | --- | --- |
|  | **L Par** | **R Par** | **L IFG** | **R IFG** | **L NFA** | **R NFA** | **L Par + NFA** | **R Par + NFA** |
| Collapsed across tasks and averaged over direction | 7.47* | 4.46* | 3.34* | 1.42 | 0.77 | -0.18 | 4.65* | 1.20 |
| Collapsed across tasks, nonsymbolic to symbolic | 5.52* | 3.48* | 2.47 | 0.81 | 1.06 | -0.28 | 4.23* | 1.45 |
| Collapsed across tasks, symbolic to nonsymbolic | 7.12* | 3.85* | 3.11* | 1.42 | 0.16 | -0.03 | 3.26* | 0.54 |
| Identify,  nonsymbolic to symbolic | 5.90* | 2.78* | 1.66 | 0.91 | 0.52 | -2.25 | 3.16* | 1.46 |
| Identify,  symbolic to nonsymbolic | 6.19* | 4.26* | 0.83 | 0.60 | 0.57 | 0.49 | 1.94 | 1.49 |
| Compare,  nonsymbolic to symbolic | 3.05* | 0.14 | 0.84 | 0.18 | 0.17 | 1.35 | 1.00 | 0.97 |
| Compare,  symbolic to nonsymbolic | 4.85* | 0.89 | 0.78 | 0.81 | 0.81 | -0.12 | 2.23 | 0.88 |

* *p*  < .05 Bonferroni adjusted for testing across 8 ROIs

| **Supplementary Table S13.** Generalization Accuracy for Identify to Compare | | | | | | | | | | | | | | | | | | |
| --- | --- | --- | --- | --- | --- | --- | --- | --- | --- | --- | --- | --- | --- | --- | --- | --- | --- | --- |
|  |  |  | |  |  |  |  |  |  |  |  |  |  |  |  |  |  |  |
|  | | **L Par** | | | **R Par** | | **L IFG** | | **R IFG** | | **L NFA** | | **R NFA** | | **L Par + NFA** | | **R Par + NFA** | |
| *t* | | 4.75 | |  | 4.20 |  | 4.46 |  | 1.83 |  | 0.81 |  | 0.27 |  | 1.93 |  | 0.77 |  |
| *p* | | | < .001 |  | .001 |  | < .001 |  | 0.606 |  | 1.000 |  | 1.000 |  | 0.485 |  | 1.000 |  |
| Mean |  | 0.277 | |  | 0.274 |  | 0.273 |  | 0.262 |  | 0.254 |  | 0.251 |  | 0.260 |  | 0.254 |  |
| SD |  | 0.0356 | |  | 0.0356 |  | 0.0328 |  | 0.0416 |  | 0.0344 |  | 0.0331 |  | 0.0310 |  | 0.0310 |  |
| *Note*. p-values are Bonferroni adjusted for testing across 8 ROIs | | | | | | | | | | | | | | | | | | |

| **Supplementary Table S14.** Generalization Accuracy for Compare to Identify | | | | | | | | | | | | | | | | | |
| --- | --- | --- | --- | --- | --- | --- | --- | --- | --- | --- | --- | --- | --- | --- | --- | --- | --- |
|  |  |  |  |  |  |  |  |  |  |  |  |  |  |  |  |  |  |
|  | | **L Par** | | **R Par** | | **L IFG** | | **R IFG** | | **L NFA** | | **R NFA** | | **L Par + NFA** | | **R Par +**  **NFA** | |
| *T* |  | 5.19 |  | 5.29 |  | 2.41 |  | 3.45 |  | 1.66 |  | 1.06 |  | 3.61 |  | 2.77 |  |
| *P* | | < .001 |  | < .001 |  | .168 |  | 0.011 |  | 0.839 |  | 1.000 |  | 0.007 |  | 0.069 |  |
| Mean |  | 0.281 |  | 0.274 |  | 0.262 |  | 0.267 |  | 0.258 |  | 0.257 |  | 0.270 |  | 0.265 |  |
| SD |  | 0.0370 |  | 0.0280 |  | 0.0307 |  | 0.0313 |  | 0.0313 |  | 0.0397 |  | 0.0346 |  | 0.0336 |  |
| *Note*. p-values are Bonferroni adjusted for testing across 8 ROIs | | | | | | | | | | | | | | | | | |

| **Supplementary Table S15.** Generalization Accuracy for Nonsymbolic: Identify to Compare | | | | | | | | | | | | | | | | | |
| --- | --- | --- | --- | --- | --- | --- | --- | --- | --- | --- | --- | --- | --- | --- | --- | --- | --- |
|  |  |  |  |  |  |  |  |  |  |  |  |  |  |  |  |  |  |
|  | | **L Par** | | **R Par** | | **L IFG** | | **R IFG** | | **L NFA** | | **R NFA** | | **L Par + NFA** | | **L Par + NFA** | |
| Mean | | 0.289 |  | 0.294 |  | 0.274 |  | 0.270 |  | 0.260 |  | 0.254 |  | 0.272 |  | 0.265 |  |
| SD | | 0.0606 |  | 0.0592 |  | 0.0462 |  | 0.0491 |  | 0.0561 |  | 0.0468 |  | 0.0432 |  | 0.0387 |  |
| *t* | | 4.06 |  | 4.63 |  | 3.25 |  | 2.57 |  | 1.11 |  | 0.56 |  | 3.20 |  | 2.43 |  |
| *p_bonferroni_* | | .002 |  | <.001 |  | .019 |  | .115 |  | 1.000 |  | 1.000 |  | .022 |  | .159 |  |

| **Supplementary Table S16.** Generalization Accuracy for Nonsymbolic: Compare to Identify | | | | | | | | | | | | | | | | | |
| --- | --- | --- | --- | --- | --- | --- | --- | --- | --- | --- | --- | --- | --- | --- | --- | --- | --- |
|  |  |  |  |  |  |  |  |  |  |  |  |  |  |  |  |  |  |
|  | | **L Par** | | **R Par** | | **L IFG** | | **R IFG** | | **L NFA** | | **R NFA** | | **L Par + NFA** | | **R Par + NFA** | |
| Mean | | 0.301 |  | 0.301 |  | 0.284 |  | 0.281 |  | 0.272 |  | 0.260 |  | 0.274 |  | 0.278 |  |
| SD | | 0.0612 |  | 0.0486 |  | 0.0377 |  | 0.0500 |  | 0.0412 |  | 0.0473 |  | 0.0503 |  | 0.0511 |  |
| *t* | | 5.20 |  | 6.58 |  | 5.63 |  | 3.88 |  | 3.35 |  | 1.27 |  | 2.99 |  | 3.36 |  |
| *p_bonferroni_* | | <.001 |  | <.001 |  | <.001 |  | .003 |  | .015 |  | 1.00 |  | .039 |  | .014 |  |

| **Supplementary Table S17.** Generalization Accuracy for Symbolic: Identify to Compare | | | | | | | | | | | | | | | | | |
| --- | --- | --- | --- | --- | --- | --- | --- | --- | --- | --- | --- | --- | --- | --- | --- | --- | --- |
|  |  |  |  |  |  |  |  |  |  |  |  |  |  |  |  |  |  |
|  | | **L Par** | | **R Par** | | **L IFG** | | **R IFG** | | **L NFA** | | **R NFA** | | **L Par + NFA** | | **R Par + NFA** | |
| Mean | | 0.279 |  | 0.287 |  | 0.248 |  | 0.260 |  | 0.257 |  | 0.258 |  | 0.264 |  | 0.267 |  |
| SD | | 0.0502 |  | 0.0420 |  | 0.0638 |  | 0.0487 |  | 0.0425 |  | 0.0525 |  | 0.0588 |  | 0.0489 |  |
| *t* | | 3.55 |  | 5.43 |  | -0.16 |  | 1.31 |  | 1.08 |  | 0.92 |  | 1.46 |  | 0.86 |  |
| *p_bonferroni_* | | .008 |  | <.001 |  | 1.000 |  | 1.000 |  | 1.000 |  | 1.000 |  | 1.000 |  | 1.000 |  |

| **Supplementary Table S18.** Generalization Accuracy for Nonsymbolic: Compare to Identify | | | | | | | | | | | | | | | | | |
| --- | --- | --- | --- | --- | --- | --- | --- | --- | --- | --- | --- | --- | --- | --- | --- | --- | --- |
|  |  |  |  |  |  |  |  |  |  |  |  |  |  |  |  |  |  |
|  | | **L Par** | | **R Par** | | **L IFG** | | **R IFG** | | **L NFA** | | **R NFA** | | **L Par + NFA** | | **R Par + NFA** | |
| Mean | | 0.278 |  | 0.273 |  | 0.241 |  | 0.253 |  | 0.249 |  | 0.250 |  | 0.260 |  | 0.266 |  |
| SD | | 0.0598 |  | 0.0514 |  | 0.0516 |  | 0.0588 |  | 0.0567 |  | 0.0397 |  | 0.0448 |  | 0.0611 |  |
| *t* | | 2.95 |  | 2.84 |  | -1.05 |  | 0.34 |  | -0.46 |  | -0.05 |  | 1.38 |  | 1.64 |  |
| *p_bonferroni_* | | .044 |  | .057 |  | 1.000 |  | 1.000 |  | 1.000 |  | 1.000 |  | 1.00 |  | .877 |  |

| **Supplementary Table S19.** Task generalization accuracy across all analyses. Numbers refer to t-statistic values from the comparison of mean classification across numerosities compared to chance (25%). | | | | | | | | |
| --- | --- | --- | --- | --- | --- | --- | --- | --- |
|  | **L Par** | **R Par** | **L IFG** | **R IFG** | **L NFA** | **R NFA** | **L Par + NFA** | **R Par + NFA** |
| Collapsed across formats and averaged over direction | 6.48* | 5.87* | 4.87* | 3.37 * | 1.74 | 1.06 | 3.46* | 2.42* |
| Collapsed across formats, identify to compare | 4.75* | 4.20* | 4.46* | 1.83 | 0.81 | 0.27 | 1.93 | 0.77 |
| Collapsed across formats, compare to identify | 5.19* | 5.29* | 2.41 | 3.45* | 1.66 | 1.06 | 3.61* | 2.77 |
| Nonsymbolic,  identify to compare | 4.06* | 4.63* | 3.25* | 2.57 | 1.11 | 0.56 | 3.20* | 2.43 |
| Nonsymbolic,  compare to identify | 5.20* | 6.58* | 5.63* | 3.88* | 3.35* | 1.27 | 2.99* | 3.36* |
| Symbolic,  identify to compare | 3.55* | 5.43* | -0.16 | 1.31 | 1.08 | 0.92 | 1.46 | 0.86 |
| Symbolic,  compare to identify | 2.95* | 2.84* | -1.05 | 0.34 | -0.46 | -0.05 | 1.38 | 1.64 |

* *p*  < .05 Bonferroni adjusted for testing across 8 ROIs

**Supplementary Table S20.** Correlations between decoding accuracy rates and measures of math achievement.

| **Decoding Accuracy Rates** |  | Math Achievement^2^ | Math  Fluency | Math  Calculation |
| --- | --- | --- | --- | --- |
| L Parietal (symbolic) | Pearson *r* | -0.012 | -0.004 | -0.011 |
|  | p-value | 0.944 | 0.980 | 0.984 |
|  |  |  |  |  |
| L Parietal (nonsymbolic) | Pearson *r* | -0.090 | 0.047 | -0.122 |
|  | p-value | 0.584 | 0.778 | 0.461 |
|  |  |  |  |  |
| R Parietal (symbolic) | Pearson *r* | -0.023 | -0.119 | 0.041 |
|  | p-value | 0.889 | 0.471 | 0.806 |
|  |  |  |  |  |
| R Parietal (nonsymbolic) | Pearson *r* | 0.185 | -0.082 | 0.257 |
|  | p-value | 0.259 | 0.622 | 0.114 |
|  |  |  |  |  |
| L IFG (symbolic) | Pearson *r* | 0.179 | 0.173 | 0.144 |
|  | p-value | 0.276 | 0.292 | 0.381 |
|  |  |  |  |  |
| L IFG (nonsymbolic) | Pearson *r* | -0.062 | -0.045 | -0.044 |
|  | p-value | 0.707 | 0.788 | 0.792 |
|  |  |  |  |  |
| R IFG (symbolic) | Pearson *r* | -0.055 | -0.002 | -0.062 |
|  | p-value | 0.739 | 0.989 | 0.706 |
|  |  |  |  |  |
| R IFG (nonsymbolic) | Pearson *r* | -0.076 | -0.179 | -0.015 |
|  | p-value | 0.646 | 0.275 | 0.926 |
|  |  |  |  |  |
| L NFA (symbolic) | Pearson *r* | 0.198 | 0.026 | 0.226 |
|  | p-value | 0.227 | 0.875 | 0.166 |
|  |  |  |  |  |
| L NFA (nonsymbolic) | Pearson *r* | 0.255 | 0.035 | 0.292 |
|  | p-value | 0.092 | 0.833 | 0.072 |
|  |  |  |  |  |
| R NFA (symbolic) | Pearson *r* | 0.131 | 0.183 | 0.090 |
|  | p-value | 0.427 | 0.266 | 0.586 |
|  |  |  |  |  |
| R NFA (nonsymbolic) | Pearson *r* | -0.092 | -0.188 | -0.24 |
|  | p-value | 0.578 | 0.251 | 0.882 |
|  |  |  |  |  |

**Supplementary Tables S21: Distance Effect Mixed Model for Targets 4 and 6 in Left Parietal ROI.**

# Mixed Model

| Model Info | | | | |  |  |
| --- | --- | --- | --- | --- | --- | --- |
|  |  |  | |  | |  |
| **Info** |  | | | |  |  |
| Estimate |  | | Linear mixed model fit by REML | |  | |
| Call |  | | AccuracyRate ~ 1 + Distance + Button+( 1 + Distance + Button \| Subject ) | |  | |
| AIC |  | | -762.038 | |  | |
| R-squared Marginal |  | | 0.264 | |  | |
| R-squared Conditional |  | | 0.380 | |  | |
|  | | | | |  |  |

## Model Results

| Fixed Effect Omnibus tests | | | | | | | | | |
| --- | --- | --- | --- | --- | --- | --- | --- | --- | --- |
|  |  |  |  |  |  |  |  |  |  |
|  | | **F** | | **Num df** | | **Den df** | | **p** | |
| Distance |  | 53.330 |  | 1 |  | 38.0 |  | < .001 |  |
| Button |  | 0.319 |  | 1 |  | 38.0 |  | 0.573 |  |
|  | | | | | | | | | |

| Fixed Effects Parameter Estimates | | | | | | | | | | | | | | | |
| --- | --- | --- | --- | --- | --- | --- | --- | --- | --- | --- | --- | --- | --- | --- | --- |
|  | | | | | | **95% Confidence Interval** | | | |  | | | | | |
| **Names** | | **Estimate** | | **SE** | | **Lower** | | **Upper** | | **df** | | **t** | | **p** | |
| (Intercept) |  | 0.30353 |  | 0.01465 |  | 0.2748 |  | 0.3322 |  | 57.5 |  | 20.713 |  | < .001 |  |
| Distance |  | -0.02989 |  | 0.00409 |  | -0.0379 |  | -0.0219 |  | 38.0 |  | -7.303 |  | < .001 |  |
| Button |  | 0.00417 |  | 0.00738 |  | -0.0103 |  | 0.0186 |  | 38.0 |  | 0.565 |  | 0.573 |  |
|  | | | | | | | | | | | | | | | |

| Random Components | | | | | | | | | |
| --- | --- | --- | --- | --- | --- | --- | --- | --- | --- |
|  |  |  |  |  |  |  |  |  |  |
| **Groups** | | **Name** | | **SD** | | **Variance** | | **ICC** | |
| Subject |  | (Intercept) |  | 0.04534 |  | 0.00206 |  | 0.328 |  |
|  |  | Distance |  | 0.01975 |  | 3.90e-4 |  |  |  |
|  |  | Button |  | 0.00390 |  | 1.52e-5 |  |  |  |
| Residual |  |  |  | 0.06491 |  | 0.00421 |  |  |  |
|  | | | | | | | | | |

| Random Parameters correlations | | | | | | | |
| --- | --- | --- | --- | --- | --- | --- | --- |
|  |  |  |  |  |  |  |  |
| **Groups** | | **Param.1** | | **Param.2** | | **Corr.** | |
| Subject |  | (Intercept) |  | Distance |  | -1.000 |  |
|  |  | (Intercept) |  | Button |  | -1.000 |  |
|  |  | Distance |  | Button |  | 1.000 |  |
|  | | | | | | | |

## Effects Plots


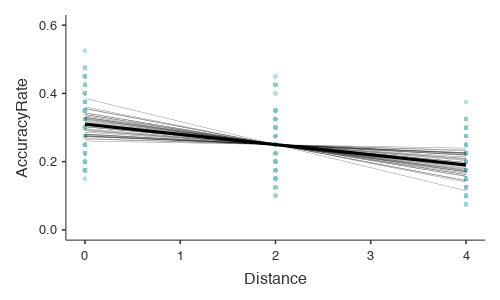


Note: Random effects are plotted by Subject

**Supplementary Tables S22: Distance Effect Mixed Model for Targets 4 and 6 in Right Parietal ROI.**

# Mixed Model

| Model Info | | | | |  |  |
| --- | --- | --- | --- | --- | --- | --- |
|  |  |  | |  | |  |
| **Info** |  | | | |  |  |
| Estimate |  | | Linear mixed model fit by REML | |  | |
| Call |  | | AccuracyRate ~ 1 + Distance + Button+( 1 + Distance + Button \| Subject ) | |  | |
| AIC |  | | -735.632 | |  | |
| R-squared Marginal |  | | 0.115 | |  | |
| R-squared Conditional |  | | 0.230 | |  | |
|  | | | | |  |  |

## Model Results

| Fixed Effect Omnibus tests | | | | | | | | | |
| --- | --- | --- | --- | --- | --- | --- | --- | --- | --- |
|  |  |  |  |  |  |  |  |  |  |
|  | | **F** | | **Num df** | | **Den df** | | **p** | |
| Distance |  | 21.583 |  | 1 |  | 38.0 |  | < .001 |  |
| Button |  | 0.238 |  | 1 |  | 38.0 |  | 0.628 |  |
|  | | | | | | | | | |

| Fixed Effects Parameter Estimates | | | | | | | | | | | | | | | |
| --- | --- | --- | --- | --- | --- | --- | --- | --- | --- | --- | --- | --- | --- | --- | --- |
|  | | | | | | **95% Confidence Interval** | | | |  | | | | | |
| **Names** | | **Estimate** | | **SE** | | **Lower** | | **Upper** | | **df** | | **t** | | **p** | |
| (Intercept) |  | 0.28141 |  | 0.01458 |  | 0.2528 |  | 0.3100 |  | 42.8 |  | 19.295 |  | < .001 |  |
| Distance |  | -0.01859 |  | 0.00400 |  | -0.0264 |  | -0.0107 |  | 38.0 |  | -4.646 |  | < .001 |  |
| Button |  | 0.00385 |  | 0.00788 |  | -0.0116 |  | 0.0193 |  | 38.0 |  | 0.488 |  | 0.628 |  |
|  | | | | | | | | | | | | | | | |

| Random Components | | | | | | | | | |
| --- | --- | --- | --- | --- | --- | --- | --- | --- | --- |
|  |  |  |  |  |  |  |  |  |  |
| **Groups** | | **Name** | | **SD** | | **Variance** | | **ICC** | |
| Subject |  | (Intercept) |  | 0.03635 |  | 0.00132 |  | 0.221 |  |
|  |  | Distance |  | 0.01827 |  | 3.34e-4 |  |  |  |
|  |  | Button |  | 0.00970 |  | 9.41e-5 |  |  |  |
| Residual |  |  |  | 0.06819 |  | 0.00465 |  |  |  |
|  | | | | | | | | | |

| Random Parameters correlations | | | | | | | |
| --- | --- | --- | --- | --- | --- | --- | --- |
|  |  |  |  |  |  |  |  |
| **Groups** | | **Param.1** | | **Param.2** | | **Corr.** | |
| Subject |  | (Intercept) |  | Distance |  | -0.920 |  |
|  |  | (Intercept) |  | Button |  | -0.187 |  |
|  |  | Distance |  | Button |  | -0.212 |  |
|  | | | | | | | |

## Effects Plots


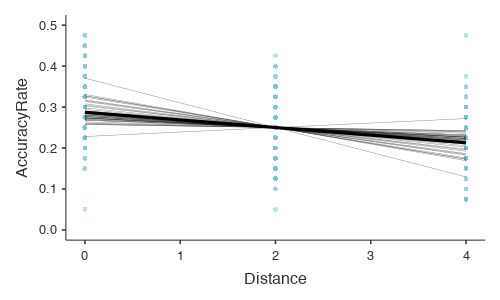


Note: Random effects are plotted by Subject

| **Supplementary Table 23.** Correlations between math achievement and behavioral number comparison task performance. | | | | |
| --- | --- | --- | --- | --- |
|  |  | **Math Composite** | **Calculation** | **Math Fluency** |
| Symbolic Comparison *P* | Pearson's r | -0.232 | -0.131 | -0.433** |
|  | p-value | 0.155 | 0.426 | 0.006 |
| Nonsymbolic Comparison *P* | Pearson's r | 0.102 | 0.051 | 0.155 |
|  | p-value | 0.536 | 0.758 | 0.345 |
| Nonsymbolic Comparison *w* | Pearson's r | 0.071 | 0.053 | 0.027 |
|  | p-value | 0.667 | 0.750 | 0.869 |
| Note. * p < .05, ** p < .01, *** p < .001 | | | | |
|  | | | | |
